# DU-KAN: A Dual-Domain Enhanced Deep-Learning Framework for Digital Pathology-Based Oxford MEST-C Assessment in Pediatric IgA Nephropathy

**DOI:** 10.3390/bioengineering13101191

**Published:** 2026-10-13

**Authors:** Jingzhi Fu, Hengzhi Cui, Yihui Zhu, Tiansheng Gao, Pei Zhang, Chunlin Gao

**Affiliations:** 1 School of Automation, Nanjing University of Information Science and Technology, Nanjing 210044, China; 001822@nuist.edu.cn (J.F.); 202412492674@nuist.edu.cn (H.C.); zhuyh90@nuist.edu.cn (Y.Z.); 202413360074@nuist.edu.cn (T.G.); 2 Jiangsu Collaborative Innovation Center of Atmospheric Environment and Equipment Technology, Nanjing 210044, China; 3 Department of Pediatrics, Jinling Hospital, Affiliated Hospital of Medical School, Nanjing University, Nanjing 210002, China

**Keywords:** pediatric IgA nephropathy, digital pathology, histopathological image segmentation, deep learning, U-KAN, Oxford MEST-C classification, computer-aided diagnosis

## Abstract

Oxford MEST-C assessment of pediatric immunoglobulin A nephropathy (IgAN) relies on pathologists’ interpretation of complex renal biopsy structures. Automated segmentation may support standardized quantification but is challenged by scale variation, weak boundaries, and small lesions. We developed DU-KAN, a stage-adapted U-KAN framework combining a shallow convolution–MaxViT dual-branch encoder with adapted heterogeneous feature fusion and wavelet–prototype skip enhancement. A single-center dataset of 532 periodic acid–Schiff-stained images from 278 children was partitioned at the patient level. On the held-out test set of 55 patients, DU-KAN achieved a mean Dice of 69.58% across eight classes, including background (95% patient-level bootstrap CI, 66.23–71.91), exceeding U-KAN by 3.56 percentage points (95% CI, 1.90–5.26). Foreground mean Dice was 65.42%. On the Synapse abdominal CT benchmark, mean Dice across eight organs was 87.21%, supporting cross-task architectural generalization, rather than external validation in pediatric IgAN. Segmentation outputs were further aggregated into case-level MEST-C-related indicators. In an exploratory 40-case evaluation, complete agreement across the five assessed indicators was 65.00% (26/40), while sensitivity was 37.50% for E (3/8) and 50.00% for T (1/2). The 97.50% agreement for T largely reflected the predominance of negative cases and did not establish reliable positive-case detection. DU-KAN improves the internal segmentation performance and achieves structured pathological quantification, which can provide auxiliary reference for clinical judgment of renal pathologists.

## 1. Introduction

IgA nephropathy (IgAN) in children is one of the most common primary glomerular diseases in childhood. The diagnosis of IgAN mainly depends on renal biopsy and immunopathology [1]. With the development of renal pathology, Roberts et al. [2] proposed the Oxford classification system to standardize the histological changes of IgAN from four dimensions: mesangial cell proliferation (M), endocapillary cell proliferation (E), segmental glomerulosclerosis or adhesion (S), and tubular atrophy/interstitial fibrosis (T). Subsequently, Trimarchi et al. [3] introduced crescent score (C) on this basis to form a more complete pathological evaluation system of MEST-C.

The MEST-C system covers multiple structural levels such as glomeruli and tubulointerstitium, but the corresponding anatomical units of each index are not completely consistent. M mainly reflects the degree of cell proliferation in the mesangial area and the proportion of involved glomeruli. E and S mainly describe the pathological changes inside a single glomerulus. T reflects the structural damage in the renal cortex. C needs to count the proportion of glomeruli containing crescents. Therefore, pathologists need to integrate information from multiple glomeruli and different cortical regions to form case-level judgments in practical assessments. The study by Howie et al. [4] showed that there were differences in inter-observer agreement of different MEST-C measures, and the interpretation of some active lesions was still somewhat subjective. PAS staining can clearly show the mesangial area, capillary loops and cell proliferation, as well as sclerosis foci and tubulointerstitial changes, which is an important basis for light microscopic pathological analysis of IgAN. Therefore, the structured segmentation and quantitative analysis of PAS staining images are expected to provide more stable and reviewable auxiliary evidence for the pathological evaluation of IgAN in children.

From a computational perspective, MEST-C assessment requires the simultaneous representation of pathological evidence at four distinct structural levels. Cellular-level assessment depends on preserving fine morphological information from mesangial and endocapillary cellular components; lesion-level assessment requires accurate delineation of small, irregular, and often low-contrast sclerosis and crescent regions; glomerular-level assessment requires sufficient spatial context to establish lesion-to-glomerulus attribution and quantify the proportion of affected glomeruli; and cortical-level assessment requires estimation of tissue involvement over a substantially larger spatial extent. These requirements differ not only in object size, but also in the type and range of spatial context needed for interpretation. Fine cellular components and focal lesions depend strongly on high-resolution local information, whereas glomerular attribution and cortical involvement require broader receptive fields and long-range contextual modeling. A single conventional representation is therefore difficult to optimize for all four levels simultaneously: progressive downsampling facilitates semantic abstraction and contextual modeling, but may attenuate small-target responses, weak boundaries, and fine morphological details.

In terms of methodology, deep-learning technology has been widely used in medical image segmentation in recent years. Ronneberger et al. [5] proposed U-Net to achieve multi-scale feature fusion through encoder–decoder structure and jump connection, which laid the basic framework for medical image segmentation. On this basis, Oktay et al. [6] proposed Attention U-Net, which uses the attention-gating mechanism to enhance the response of the target area and suppress background interference. Chen et al. [7] proposed TransUNet and introduced Transformer into the U-shaped network to enhance the modeling ability of the model for long-range dependencies. Tu et al. [8] proposed MaxViT to jointly model local features and global features through block attention and grid attention, which showed strong feature-expression ability in a variety of visual tasks. In addition, Li et al. [9] proposed U-KAN, introducing the Kolmogorov–Arnold Network [10] into the deep-feature modeling process of the U-shaped structure, and using learnable nonlinear functions to enhance the expression ability of the model, which achieved a good balance between performance and efficiency in the task of medical image segmentation. The above methods improve the feature expression ability of medical image segmentation models from different perspectives, and provide an important technical basis for the fine analysis of complex pathological structures.

In the field of renal computational pathology, researchers have carried out a lot of exploration around the segmentation of tissue structure and the analysis of pathological features. Kannan et al. [11] realized the detection and segmentation of glomeruli, which can be used to count the number of glomeruli and extract their contour. Hermsen et al. [12] performed multi-category segmentation and quantitative evaluation of PAS-stained renal tissue. Bouteldja et al. [13] constructed a multi-class segmentation model to realize the joint recognition of structures such as glomeruli, tubules, and blood vessels. Jayapandian et al. [14] further proposed cross-staining and cross-center segmentation methods for renal cortical structure, which improved the generalization ability of the model.

In IgAN-related studies, Jaugey et al. [15] proposed a MEST-C automatic scoring framework based on lesion segmentation, which verified the feasibility of generating case-level scores from lesion segmentation. Altini et al. [16] combined glomerular instance segmentation with Oxford classification to achieve the prediction of MEST-related indicators at the glomerular level. In addition, Testa et al. [17] constructed IgAN prognostic prediction models based on PAS images, which verified the application potential of pathological images in risk assessment. Cho et al. [18] used multicenter data to classify glomerular lesions and predict disease progression. Pan et al. [19] proposed a multi-task deep network to jointly model lesion recognition, lesion classification and prognosis prediction. In general, the existing research has made important progress in the segmentation of renal tissue structure and the auxiliary analysis of IgAN.

Previous renal segmentation studies have demonstrated the feasibility of delineating multiple anatomical structures, while segmentation-based IgAN frameworks have already linked image-derived measurements to MEST-C assessment. The challenge addressed in this study is to coordinate fine cellular morphology, focal lesion boundaries, and broader tissue context within a shared segmentation architecture. These requirements place competing demands on spatial resolution and contextual coverage, while subsequent case-level assessment requires explicit anatomical attribution and aggregation. Direct score-prediction approaches may, additionally. lack explicit spatial evidence, although this limitation does not apply to all segmentation-based frameworks.

These limitations directly motivate the four principal components of our framework. First, the competing requirements of fine-cellular- and lesion-detail preservation and broader tissue-context modeling within the available image fields motivate the convolution–MaxViT dual-branch encoder (DAB). The convolutional branch retains local inductive bias for texture, morphology, and boundary representation, whereas the MaxViT branch supplements cross-region and long-range contextual relationships at high-resolution encoding stages. Second, because convolutional and attention-based features exhibit different scale- and location-dependent responses to cells, lesions, glomerular boundaries, and larger tissue regions, simple addition or channel concatenation may not adequately integrate these heterogeneous representations. We therefore introduce heterogeneous multi-kernel coordinate recalibration fusion (HMCRF) to perform multi-scale morphological modeling, position-sensitive recalibration, and selective integration of the two feature sources. Third, repeated downsampling can attenuate weak boundaries, directional textures, and responses from small or low-contrast structures, particularly crescents, sclerosis foci, mesangial cells, and endothelial cells. Wavelet–prototype adaptive query fusion (WPAQF) is therefore introduced in the deep-skip pathways to combine frequency-domain boundary information with prototype-based semantic representations and compensate for this information loss. Finally, pixel-level segmentation alone does not establish the anatomical evaluation units required for case-level MEST-C assessment. Segmentation-derived measurements are, therefore, organized by relevant tissue compartments and combined using prespecified aggregation rules to generate reviewable MEST-C-related indicators. Contextual modeling is restricted to the available image fields; whole-slide cortical coverage remains outside the current validation scope.

The proposed framework builds on several established mechanisms, rather than treating all underlying operations as newly introduced components. U-KAN provides the backbone architecture, MaxViT provides block- and grid-based contextual modeling, multi-kernel depthwise convolution provides multi-scale local representation, coordinate attention provides direction-aware spatial recalibration, and wavelet decomposition, prototype-based attention, and squeeze-and-excitation provide complementary frequency-domain, semantic, and channel-wise representations. These individual mechanisms are not claimed as methodological novelties of this study. Instead, the contribution lies in their stage-specific redesign and integration for multilevel renal pathological representation. Specifically, DAB constitutes the primary newly designed architectural component by introducing a parallel MaxViT auxiliary pathway into the shallow U-KAN encoder; HMCRF improves existing multi-kernel and coordinate-recalibration mechanisms for heterogeneous convolution–MaxViT feature fusion; and WPAQF adapts the wavelet–prototype query framework to deep-skip pathways, incorporating MB-SaE-based channel recalibration after query summation and before query self-attention.

Based on this rationale, we developed Dual-Domain Enhanced U-KAN (DU-KAN) for multi-structure segmentation of pediatric IgAN PAS images, and constructed an annotated dataset with seven foreground classes and one background class. The main contributions are summarized as follows.

(1) A convolution–MaxViT dual-branch encoder (DAB) is newly designed for the shallow high-resolution stages of U-KAN. The original convolutional pathway is retained for local morphological representation, while a parallel MaxViT branch is introduced for cross-region contextual modeling. The two branches remain complementary heterogeneous representations before fusion.
(2) Building on existing multi-kernel convolution and coordinate-attention mechanisms, an improved HMCRF is developed for convolution–MaxViT heterogeneous feature fusion. Its methodological modification lies in integrating feature alignment, multi-scale morphological modeling, coordinate-sensitive recalibration, and residual projection into a unified shallow-skip fusion process.
(3) WPAQF adapts an established wavelet–prototype query framework to the E4–E5 deep-skip pathways. MB-SaE-based channel recalibration is applied after query summation and before query self-attention, while the original KAN main encoding pathway is retained.
(4) An anatomically informed quantification and case-level aggregation workflow is established to convert multi-structure segmentation outputs into reviewable MEST-C-related indicators. Prespecified rules connect image-derived measurements with the relevant anatomical evaluation units and retain intermediate evidence for case review.

## 2. Materials and Methods

### 2.1. Overall Process

**Figure 1:**
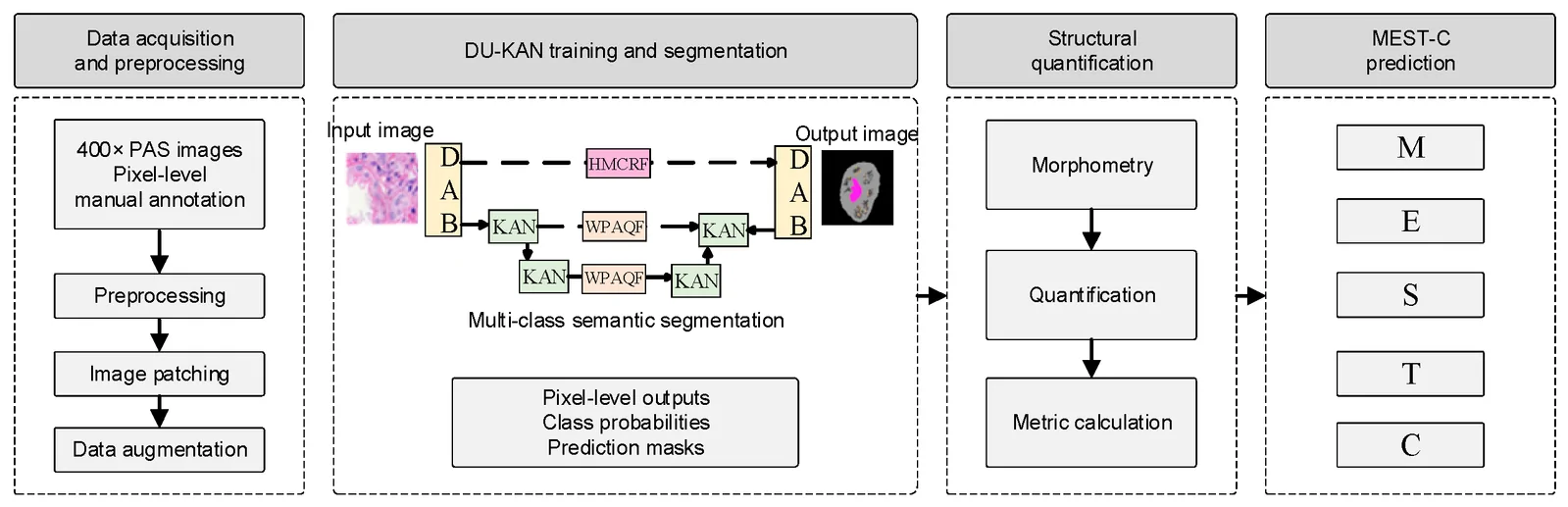
Segmentation and MEST-C determination workflow for pediatric IgAN PAS images.

As shown in Figure 1, the proposed workflow comprises four stages: (1) preprocessing and pixel-level annotation of PAS images; (2) multiclass segmentation using DU-KAN; (3) conversion of segmentation outputs into anatomically meaningful quantitative variables, including cellular burden, lesion presence/area, glomerular attribution, and tissue proportion; and (4) case-level aggregation according to the anatomical evaluation units of MEST-C. Detailed segmentation, quantification, and aggregation procedures are described in the following sections.

### 2.2. Data Set Construction, Patient-Level Division and Evaluation Scheme

#### 2.2.1. Patient-Level Division and Labeling of PAS Pathological Data Set of Children IgAN

Digital pathological images of paraffin sections of renal needle biopsies in children with IgAN, including PAS, Masson and HE staining, were provided by the pathology department of the collaborating hospitals. To reduce domain differences introduced by different staining types, segmentation experiments included only the 400× high-power field of PAS staining. The box selection and pixel labeling of the region of interest (ROI) were completed by senior pathologists, according to the unified standards, and the areas with unclear or controversial boundaries were reviewed and corrected. Finally, 532 512 × 512 pixel labeled images were obtained from 278 children with IgAN.

Data partitioning was done prior to image cropping, category balanced sampling, and online enhancement. The patient ID was used as the grouping unit, and stratified randomization was performed according to the main types of lesions. All ROIs and image blocks from the same patient and the same biopsy specimen were classified into the same subset. The training, validation and test sets contained 372, 53 and 107 images, corresponding to 195, 28 and 55 patients, respectively. There was no crossover of patients, biopsy specimens or adjacent cut blocks among the three subsets, which reduced the risk of information leakage caused by patient-specific staining, section texture, and adjacent fields of view, from the data organization level.

Aiming at the characteristics of complex tissue structure and obvious differences in lesion scale in pathological images of children with IgAN, this study established a multi-structure semantic segmentation and annotation system for the auxiliary evaluation needs of Oxford MEST-C. According to the pathological anatomical basis corresponding to different MEST-C indicators, the key tissue structures in the image were divided into seven types of foreground regions, including crescents, sclerosis foci, mesangial lesions, tubular atrophy-related areas, mesangial cells, glomerular tissue and endothelial cells. Meanwhile, background categories were set to represent non-target areas. All annotations were performed in a pixel-level single-label coding manner; that is, each pixel corresponds to only one tissue category. The areas with blurred boundary, overlapping structure or uncertain morphology between the lesion area and normal tissue were classified according to pathological-structure characteristics and Oxford MEST-C classification criteria, and further reviewed by pathologists.

Image annotation was performed by pathologists with experience in renal pathological diagnosis. During the labeling process, pathologists marked different tissue structures and pathological regions pixel-by-pixel, according to unified labeling standards. In order to reduce possible subjective differences during manual labeling, all labeling results were subjected to a strict quality control process. Areas with unclear boundaries, complex structures or differences in interpretation were reviewed by senior renal pathologists, and the final confirmation was completed based on histomorphological features. The labeling results obtained through the above process were used as the reference standard in the training and testing stages of the model to ensure the accuracy and reliability of data labeling.

#### 2.2.2. Data Preprocessing and Training Settings

Category balancing processing was applied only to the training subset. The sampling indexes of balanced_images and balanced_labels were constructed according to the occurrence of each category in the training images, to improve the extraction probability of the category images containing scarce lesions. The validation and test sets kept their original distributions and did not participate in balanced sampling. Online enhancement, in turn, consisted of unifying the image to 512 × 512 pixels; the 256 × 256 area was randomly cut, and the position was re-selected when any category accounted for more than 75%, and the maximum retry was 10 times. The image and mask were flipped horizontally, synchronously, with 0.5 probability. Luminance, contrast, saturation, and hue perturbations were performed to simulate slide, illumination, and batch differences under the same PAS staining type; finally, the samples were filled to the specified size. Mean = [0,0,0], std = [1,1,1] means that no additional mean-standard deviation normalization based on data statistics was performed.

The training set was wrapped with RepeatDataset (times = 10) [20] so that the same original image received independent random enhancement in different iterations, thereby increasing the equivalent number of observations during training. This setting does not change the original class proportion, and the class imbalance is mainly handled by the balanced sampling index in the training subset, random clipping with restricted class proportion, and cross-entropy-Dice joint loss [21].

All comparative experiments adopted fixed patient-level division and random seed, and uniformly used the AdamW optimizer [22], a poly learning-rate strategy, 20,000 parameter updates, and effective batch size = 4. In the test phase, a 256 × 256 window and a 128 × 128 step size were used for sliding window inference, and the class probabilities of the overlapping regions were averaged, to recover the full prediction map.

All experiments were conducted on a workstation equipped with four NVIDIA RTX 3090 GPUs (24 GB; NVIDIA Corporation, Santa Clara, CA, USA). The model was implemented using Python (version 3.8.20; Python Software Foundation, Wilmington, DE, USA), PyTorch (version 1.10.0; Meta AI, Menlo Park, CA, USA), CUDA (version 11.3; NVIDIA Corporation, Santa Clara, CA, USA), cuDNN (version 8.2; NVIDIA Corporation, Santa Clara, CA, USA), and MMSegmentation (version 0.29.1; OpenMMLab, Shanghai, China). The complete source code, model implementation, training configuration files, and environment specifications developed in this study are publicly available at the GitHub repository: https://github.com/joker20011214/du-kan.

#### 2.2.3. Evaluation Datasets and Indicators

The pediatric IgAN PAS pathological dataset was used to evaluate the multi-structure segmentation performance of the model in the target pathological setting. The public Synapse dataset [23] was additionally used to examine whether the architectural improvements of DU-KAN remain effective in a substantially different segmentation task involving a different imaging modality, anatomy, and target structures. Thus, the Synapse experiments were designed to assess cross-task architectural generalization, rather than external clinical or pathological validation of pediatric IgAN. Synapse contains 30 abdominal CT cases and uses the commonly adopted split of 18 cases for training and 12 cases for testing, with eight target organs: the aorta, gallbladder, left kidney, right kidney, liver, pancreas, spleen, and stomach.

The evaluation indexes included overall pixel accuracy (aAcc), average intersection ratio (mIoU), average Precision (mPrecision), average Recall (mRecall) and average Dice coefficient (mDice). For class c, TPc, FPc, and FNc represent the number of pixels of true, false-positive, and false-negative cases, respectively, and K represents the number of categories involved in the macro average calculation. The category-level overlap index, detection index and macro average index were calculated according to Equations (1)–(6).

(1) $${IoU}_{c}=\frac{{TP}_{c}}{{TP}_{c}+{FP}_{c}+{FN}_{c}}$$

(2) $${Dice}_{c}=\frac{2\;{TP}_{c}}{2\;{TP}_{c}+{FP}_{c}+{FN}_{c}}$$

(3) $${Precision}_{c}=\frac{{TP}_{c}}{{TP}_{c}+{FP}_{c}}$$

(4) $${Recall}_{c}=\frac{{TP}_{c}}{{TP}_{c}+{FN}_{c}}$$

(5) $$mM=\frac{1}{K}\sum_{c=1}^{K}M_{c}$$

(6) $$aAcc=\frac{\sum_{c=1}^{K}{TP}_{c}}{\sum_{c=1}^{K}\left({TP}_{c}+{FN}_{c}\right)}$$

The degree of regional overlap, prediction accuracy and integrity of target detection between the single-category prediction results and the manual labeling results were measured by Equations (1)–(4), where IoU and Dice were calculated by Equations (1) and (2), respectively, to evaluate the consistency between the predicted regions and the real regions. Equations (3) and (4) calculate Precision and Recall, respectively, which are used to measure the accuracy of the model prediction results and the coverage ability of the target area. Equation (5) represents the macro average calculation of different category indicators, where M takes IoU, Dice, Precision and Recall, respectively, and the corresponding average intersection ratio (mIoU), average Dice coefficient (mDice), average Precision (mPrecision) and average Recall (mRecall) are obtained. Equation (6) represents the overall pixel accuracy (aAcc), which is used to evaluate the overall accuracy of all pixel category predictions. The mDice_all_ of the pediatric IgAN dataset was the 8-class macro average of background and 7 foreground categories, and the foreground average Dice excluded the background. mDice_all_ for the Synapse dataset is the 9-class macro average of background versus eight organs, and mDice_organ_ is the Dice arithmetic average of eight organs after excluding background.

#### 2.2.4. Statistical Uncertainty Analysis

Uncertainty in internal segmentation performance was quantified using a patient-level cluster bootstrap with 5000 resamples and a fixed random seed of 20260929. In each resample, 55 patients were sampled with replacement, and all images from each selected patient were included together. Patients selected more than once contributed their images with the corresponding multiplicity.

Pixel-level confusion counts were pooled within each resample before calculating class-specific metrics and their macro averages. Overall macro averages included eight classes, whereas foreground mean Dice excluded background. The 95% confidence intervals were defined by the 2.5th and 97.5th percentiles of the bootstrap distributions. Identical patient resamples were used across models to calculate paired performance differences. Class-specific comparisons were exploratory, without adjustment for multiple comparisons. Model predictions were held fixed throughout resampling. All reported metrics were defined in all 5000 resamples.

### 2.3. DU-KAN Model Architecture

DU-KAN uses U-KAN as the backbone and introduces stage-specific modifications at three network locations. The proposed architecture intentionally contains both newly designed and adapted components. DAB represents the primary newly designed architectural modification: a MaxViT auxiliary pathway is introduced in parallel with the original convolutional pathway at the E1–E3 high-resolution encoding stages. HMCRF is an improved heterogeneous fusion module built upon multi-kernel convolution and coordinate-attention principles, and is deployed in the shallow-skip pathways to integrate convolutional and MaxViT features. WPAQF adapts the wavelet–prototype query framework to the E4–E5 skip pathways and incorporates MB-SaE-based channel recalibration after query summation and before query self-attention. Therefore, the architectural contribution of DU-KAN lies not in replacing all underlying operators, but in assigning different mechanisms to distinct representation bottlenecks across the network hierarchy.

Rather than repeatedly stacking a uniform attention mechanism at the same scale, the three components act on shallow dual-branch encoding, heterogeneous skip fusion, and deep-skip compensation, respectively.

The network retained the five-stage coding-five-stage decoding topology of U-KAN, deep KAN paths, and multiscale jump connections, with the number of base channels set to 32 and the number of output categories set to 8.

**Figure 2:**
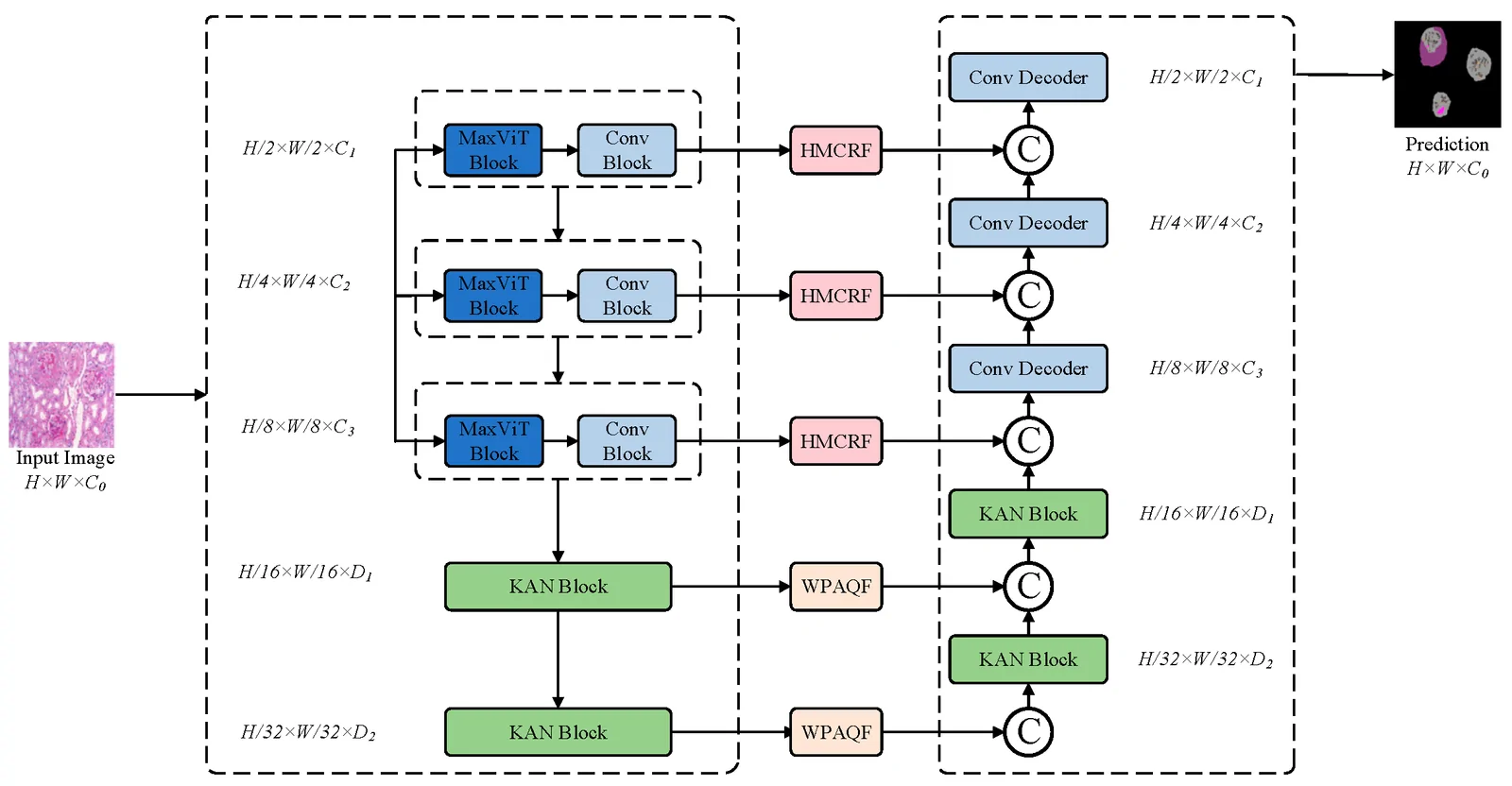
Overall architecture of DU-KAN.

The coding end consists of five stages, E1-E5, as shown in Figure 2. E1–E3 were high-resolution shallow coding stages, and the convolution main branch and MaxViT auxiliary branch were set in each stage. The convolutional main branch is responsible for step-by-step downsampling and backbone feature transfer, while the MaxViT auxiliary branch has dense interaction and cross-grid dependence in the same-scale supplementary window. After channel alignment, multi-scale local modeling, and coordinate recalibration are completed by the corresponding HMCRF, the two outputs are input D1-D3 along the shallow-jump connection. E4–E5 uses KAN blocks for deep semantic modeling, whose outputs are enhanced by WPAQF, respectively, and input D4 and D5 along two deep-jump connections. The decoding terminal is composed of five stages, D5-D1. D5 receives the deep features of E5 enhanced by WPAQF. D4 fused the upsampling feature of D5 with the jump feature of E4 enhanced by WPAQF. D3-D1 sequentially fused the upsampled features of the previous decoding stage with the fusion output of E3-E1 corresponding to HMCRF. D5-D4 uses KAN decoding blocks, and D3-D1 uses convolutional decoding blocks. D1 output was mapped into eight-class pixel probability maps by final scale recovery, 1 × 1 convolution and Softmax.

#### 2.3.1. Shallow Convolution-MaxViT Double-Branch Encoder DAB

The first three high-resolution encoding stages of the original U-KAN [9] are mainly composed of convolutional blocks, which are able to rely on local receptive fields to extract information such as stained texture, cell edge, and local morphology. However, the convolution operation essentially relies on information aggregation in the neighborhood range, and it is difficult to effectively establish the correlation relationship between different spatial locations in the shallow stage. For PAS pathological images of children with IgAN, the glomerular area contains a variety of pathological structures with obvious differences in scale and spatial distribution, such as mesangial cell proliferation, capillary loop changes, glomerular capsule wall boundaries, sclerosis foci and crescents. If only a single convolutional path is used for step-by-step coding, some weak boundary regions, sparse lesion responses and distant-tissue context information may be gradually weakened in the process of shallow-feature transfer, thereby limiting the ability of the model to distinguish fine-grained structures.

To address this limitation, we redesign the E1–E3 shallow-encoding stages of U-KAN as a convolution–MaxViT dual-branch encoder (DAB). The original convolutional pathway of U-KAN is retained as the main branch for progressive downsampling and local feature propagation, while a parallel MaxViT auxiliary branch is newly introduced at the same spatial resolution to supplement cross-region contextual information. The internal MBConv, Block Attention, and Grid Attention operations follow the established MaxViT design, and are not claimed as new attention mechanisms in this study. The architectural novelty of DAB lies in integrating MaxViT as a parallel auxiliary pathway into the shallow U-KAN encoder while preserving the original convolutional pathway.

Importantly, the two branches are not immediately merged within DAB. Instead, they are preserved as a heterogeneous feature pair at each shallow stage, and subsequently passed to HMCRF for calibrated and selective fusion. This delayed fusion design allows local convolutional representations and contextual MaxViT representations to remain complementary before cross-source recalibration.

The input feature of the l-th shallow-encoding stage is denoted as $X_{l}\in R^{H_{l}\times W_{l}\times C_{l}}$, where $H_{l}$, $W_{l}$, and $C_{l}$ represent the height, width, and number of channels of the feature map, respectively. The convolutional main branch generates local features $F_{l}^{c}$ through ${ConvStage}_{l}\left(\cdot \right)$, while the MaxViT auxiliary branch produces contextual features $F_{l}^{m}$ via ${MaxViT}_{l}\left(\cdot \right)$. The mapping relationships of the two branches are formulated as Equations (7) and (8).

(7) $$F_{l}^{c}={ConvStage}_{l}\left(X_{l}\right),\;l\in \left\{1,2,3\right\}$$

(8) $$F_{l}^{m}={MaxViT}_{l}\left(X_{l}\right),\;l\in \left\{1,2,3\right\}$$

Among them, the convolution branch mainly focused on the local details such as the internal cell structure of the glomerulus, the edge of the capillary loop and the contour of the cyst wall. The MaxViT branch enhances the information interaction between different regions through the attention mechanism, so as to supplement the spatial context beyond the local features.

The MaxViT auxiliary branch consists of a mobile inverted bottleneck convolution (MBConv) module, Block Attention, a feed-forward network, and a block attention module. FFN), Grid Attention, and FFN. Firstly, MBConv uses deep convolution and point-by-point convolution to complete local feature extraction and channel adjustment, and retains the local induction ability of the convolutional structure for pathological texture before introducing the attention mechanism. Subsequently, Block Attention divides the input features into multiple local Windows, and builds inter-pixel correlation within the Windows, so that the model can pay more attention to fine-grained structural information in continuous regions, such as the continuous boundary of the glomerular capsule wall, the local texture of the capillary plexus, and the cell distribution characteristics in the mesangial region.

**Figure 3:**
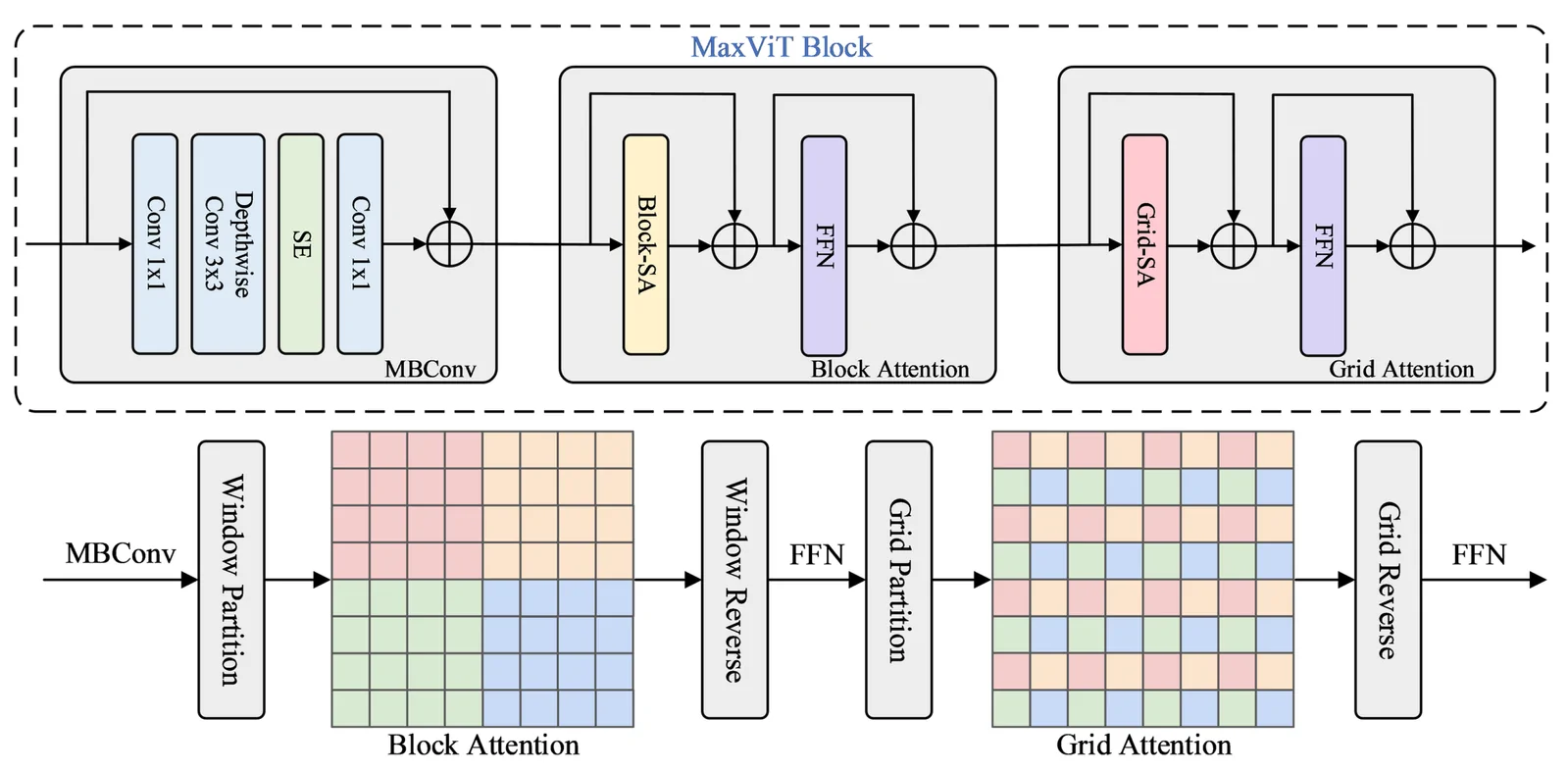
Architecture of the MaxViT auxiliary branch.

Grid Attention further reorganizes the spatial position according to the regular grid, so that features with the same grid-position relationship in different local Windows can interact, so as to establish cross-region dependence. For PAS images of children with IgAN, Grid Attention was able to enhance the association of information between scattered lesion areas, such as distant sclerotic areas, focal abnormal structures, and cellular responses at different locations. Through the synergistic effect of Block Attention and Grid Attention, MaxViT branch can obtain local fine-grained modeling ability and global spatial-relationship perception ability at the same time, which complements the convolutional branch and improves the representation ability of complex glomerular structure in the shallow stage. The MaxViT auxiliary branch structure is shown in Figure 3.

During the E1–E3 stages, DAB preserves the convolutional features $F_{l}^{c}$ and the MaxViT features $F_{l}^{m}$ at the same scale as a heterogeneous feature pair $F_{l}^{DAB}$, where $F_{l}^{DAB}$ denotes the dual-branch output of the l-th shallow-encoding stage. The convolutional features $F_{l}^{c}$ continue to propagate downward along the main encoding pathway, while the two types of features are jointly fed into the corresponding HMCRF to perform multi-scale modeling, positional calibration, and selective fusion. DAB itself does not directly perform feature fusion, and its output is defined as shown in Equation (9).

(9) $$F_{l}^{DAB}=\left(F_{l}^{c},F_{l}^{m}\right),\;\;l\in \{1,2,3\}$$

#### 2.3.2. Shallow Heterogeneous Multi-Kernel Coordinate Recalibration Fusion Module (HMCRF)

HMCRF adapts established multi-kernel convolutional designs and coordinate-attention principles to the fusion of convolutional and MaxViT features. Its study-specific design consists of organizing dual-input channel alignment, multi-kernel morphological modeling, coordinate-sensitive recalibration, and residual projection into a shallow-skip fusion pathway. The contribution concerns the configuration of these operations for heterogeneous feature integration; the underlying multi-kernel convolution and coordinate-attention principles are inherited mechanisms.

DAB outputs convolutional features and MaxViT features at the three shallow-encoding stages E1–E3. The two types of features have identical spatial resolutions, but they emphasize different representations of glomerular structures in pediatric IgA nephropathy (IgAN) PAS pathological images. Convolutional features mainly respond to local morphological information, such as glomerular-capsule-wall boundaries, capillary loop textures, and the distribution of cells in the mesangial region. In contrast, MaxViT features enhance contextual relationships among different regions through spatial relation modeling, thereby complementing information associated with focal lesions, long-range abnormal regions, and discontinuous structural patterns. Due to the differences in response regions and feature distributions between the two feature types, directly performing element-wise addition may cause mutual interference between local details and contextual information. Meanwhile, simply concatenating features along the channel dimension cannot further determine the importance of features from different sources and regions at different scales. Therefore, an adaptive fusion mechanism for heterogeneous features is required in the shallow-skip connections.

To address this issue, a Heterogeneous Multi-Kernel Coordinate Recalibration Fusion module (HMCRF) is introduced into the three shallow-skip pathways corresponding to E1–E3. The module is positioned between the DAB and the decoder. Following the procedure of heterogeneous feature alignment, multi-scale local modeling, coordinate-sensitive recalibration, and residual projection, HMCRF processes two feature maps with the same spatial scale. It selectively integrates local structural information extracted by the convolutional branch and contextual information provided by the MaxViT branch, generating enhanced skip features for the decoder to recover glomerular boundaries and internal structures.

For the l-th shallow-encoding stage, HMCRF first concatenates the outputs of the convolutional branch and the MaxViT branch along the channel dimension, and then employs a 1×1 convolution to map the two responses into a unified channel space. The channel alignment process is described in Equation (10), where the left side represents the basic fused feature. The channel expansion process is described in Equation (11), where the left side denotes the expanded feature. In the equations, l represents the index of the shallow stage, with values of 1, 2, and 3; ∥ denotes channel-wise concatenation; Conv represents the 1×1 channel alignment convolution; PWConv represents the point-wise convolution used for channel expansion; and BN and δ  denote batch normalization and the ReLU6 activation function, respectively.

(10) $$F_{l}^{0}={Conv}_{1\times 1}\left(F_{l}^{c}∥F_{l}^{m}\right)$$

(11) $$F_{l}^{e}=\delta \left(BN\left[{PWConv}_{up}\left(F_{l}^{0}\right)\right]\right)$$

Channel alignment brings the local convolutional response and cross-region context into a unified representation space, while channel expansion provides a more sufficient feature subspace for subsequent multi-kernel branching. At the same time, the basic fusion features are retained to the residual path at the end of the module, to reduce the effective information attenuation that may occur during continuous convolution and recalibration.

In the multi-scale local modeling stage, HMCRF adopts 3 × 3, 5 × 5 and 7 × 7 three-way depth convolution [24]. The smaller convolution kernel focused on the fine-grained neighborhood of mesangial cells, endothelial cells and the edge of local lesions, while the larger convolution kernel gradually expanded the effective receptive field to describe the local morphology of sclerosis foci, crescents and large tissue areas. This process is expressed as Equation (12), where DWConv represents the deep convolution of the corresponding kernel size, Shuffle represents the channel shuffling, the sum term represents the element-by-element aggregation of the three-way response, and the left end of the formula is the multi-kernel feature.

(12) $$F_{l}^{mk}=Shuffle\left\{\sum_{k\in \left\{3,5,7\right\}}\delta \left(BN\left[{DWConv}_{k\times k}\left(F_{l}^{e}\right)\right]\right)\right\}$$

The three-branch deep convolution employs kernels with different scales to capture local morphological patterns across multiple resolutions, while introducing only a marginal increase in the number of parameters. The resulting features are aggregated through element-wise summation and subsequently processed by channel shuffling, enabling effective information exchange among branches with different receptive fields and preventing multi-branch representations from being confined to isolated channel groups. Consequently, HMCRF is capable of simultaneously modeling scale variations associated with cellular-level structures, focal lesions, and larger tissue regions.

Nevertheless, multi-kernel convolution alone remains insufficient for explicitly characterizing the spatial distribution of lesion-related responses along the horizontal and vertical directions. To address this limitation, HMCRF further integrates the Recalibrated Coordinate Recalibration Attention Unit (RCRA), which independently aggregates contextual information along the width and height dimensions. By compressing one spatial dimension while preserving the positional encoding of the remaining dimension, RCRA generates direction-aware horizontal and vertical coordinate-attention weights [25]. Equation (13) describes the generation of two directional weighting maps from multi-kernel features, whereas Equation (14) illustrates the complete procedure of coordinate recalibration, dimensionality reduction projection, and residual feature fusion. Here, “⊙” denotes element-wise multiplication, and PWConv represents the point-wise convolution employed for dimensionality reduction. The term on the left-hand side of the second line in Equation (14) denotes the fusion output of HMCRF along the *l*-th shallow-skip pathway.

(13) $$\left(g_{l}^{h},g_{l}^{w}\right)=RCRA\left(F_{l}^{mk}\right)$$

(14) $$F_{l}^{HMCRF}=F_{l}^{0}+BN\left\{{PWConv}_{down}\left[F_{l}^{mk}⊙g_{l}^{h}⊙g_{l}^{w}\right]\right\}$$

**Figure 4:**
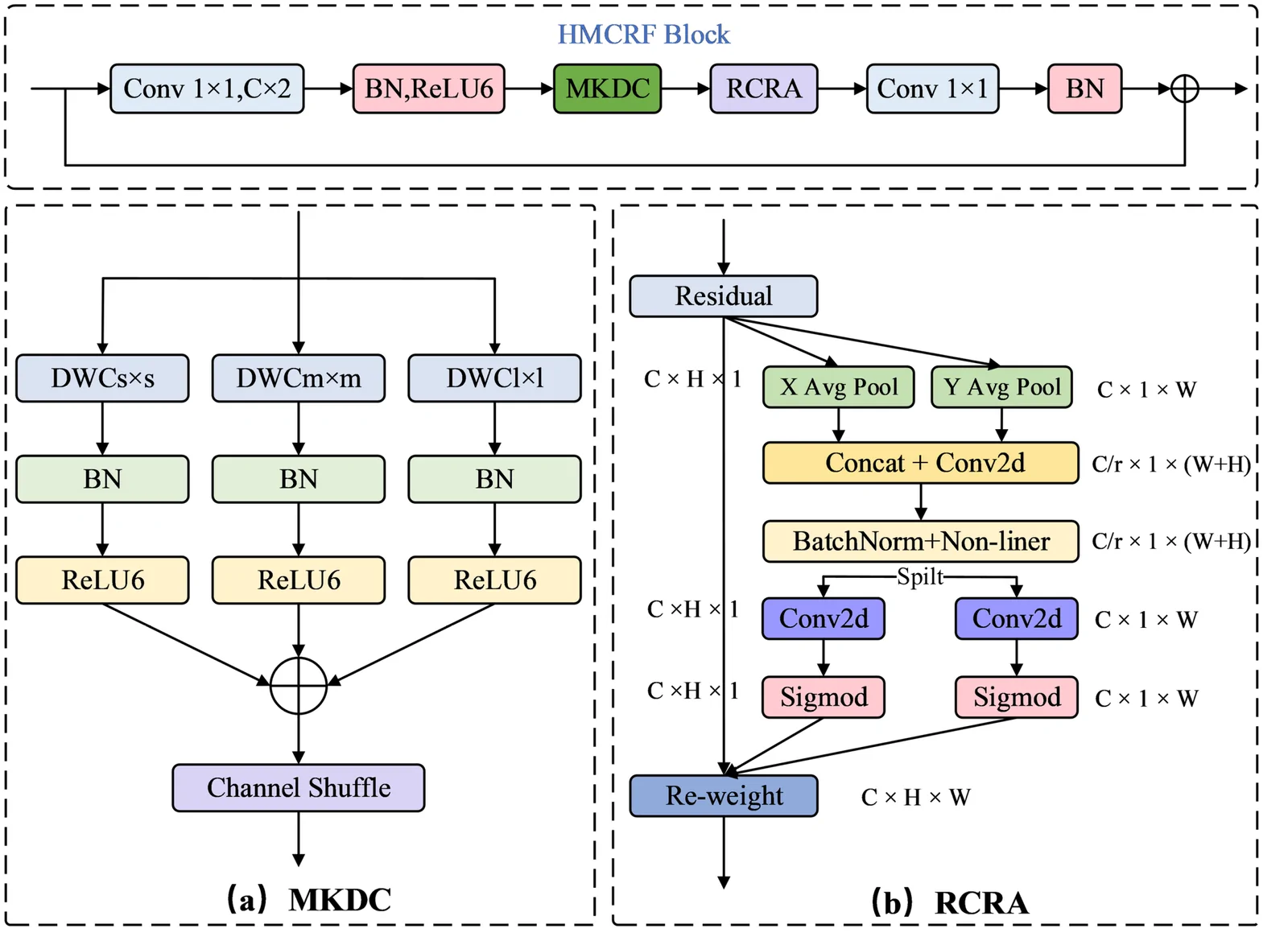
Architecture of HMCRF. (**a**) MKDC employs three multi-kernel depthwise-convolution branches; (**b**) RCRA generates coordinate-sensitive weights along the horizontal and vertical directions.

The horizontal and vertical weights can highlight locations that are consistent with the lesion contour, cell distribution, and tissue extension direction, and suppress redundant or misaligned responses in the two types of inputs, so that the convolution local information can be spatially calibrated with the MaxViT context. The residual path preserves the basic fusion information after feature alignment, and avoids the recalibration process from excessive weakening of effective details. The final output is directly fused with the upsampled features of the corresponding decoding stage. The core of HMCRF is not simply to increase the number of convolution layers, but to selectively integrate two heterogeneous information sources through multi-scale morphological modeling and coordinate-sensitive calibration. The overall structure and the multi-kernel depth convolution and coordinate recalibration substructure are shown in Figure 4.

#### 2.3.3. Deep-Jump Path Wavelet-Prototype Adaptive Query Fusion Module WPAQF

U-KAN [9] uses KAN blocks in the E4–E5 deep-coding stage to enhance the nonlinear semantic expression, but continuous downsampling will reduce the spatial resolution of the feature map, and gradually attenuate the weak boundary, fine lesion and directional texture response. Since the deep main path has already undertaken the task of semantic abstraction, it may interfere with the original KAN feature modeling if the downward propagation process is directly changed. Therefore, WPAQF with the same structure is deployed in the two deep-jump paths corresponding to E4 and E5, respectively, to implement the compensation enhancement before the deep features are fed into the decoder.

WPAQF adapts the wavelet–prototype query framework of Yan et al. [26] to the E4–E5 skip pathways of U-KAN. Wavelet decomposition, prototype-based query modeling, and MSCM-based modulation are established components of the source framework. The adaptation described here incorporates MB-SaE-based channel recalibration, drawing on SENetV2 [27], after the two query outputs are summed and before query self-attention. The resulting representation is mapped to spatial skip features for decoding, while the main KAN encoding pathway is retained.

(1) Wavelet-enhanced cross-attention (WCA)

For the deep-feature output in E4 or E5 stage, the WCA first uses Haar Discrete Wavelet Transform (DWT) for frequency decomposition, and maps the input features into one low-frequency sub-band and three directional high-frequency sub-bands. The low-frequency components mainly retain the overall contour, regional intensity distribution and large-scale semantic structure of the glomeruli, while the high-frequency components correspond to the horizontal, vertical and diagonal edge changes, which can effectively characterize the cyst wall contour, capillary texture and discontinuous boundaries of pathological regions. The decomposition process of four frequency sub-bands is shown in Equation (15). The adaptive fusion process of low-frequency structure and high-frequency details after dimension unification is shown in Equation (16). DWT represents the discrete wavelet transform, P represents the projection transform with uniform channel number and spatial size, Concat represents the high-frequency sub-band splicing, σ represents the Sigmoid function, and the two types of W represent the global and local modulation weights, respectively. The left end of the second row of the formula is the frequency domain features after modulation.

(15) $$\left(E_{i,LL},E_{i,LH},E_{i,HL},E_{i,HH}\right)=DWT\left(E_{i}\right)$$

(16) $$E_{i}^{fre}=P\left(E_{i,LL}\right)+\sigma \left(W_{i}^{g}+W_{i}^{l}\right)⊙P\left[Concat\left(E_{i,LH},E_{i,HL},E_{i,HH}\right)\right]$$

**Figure 5:**
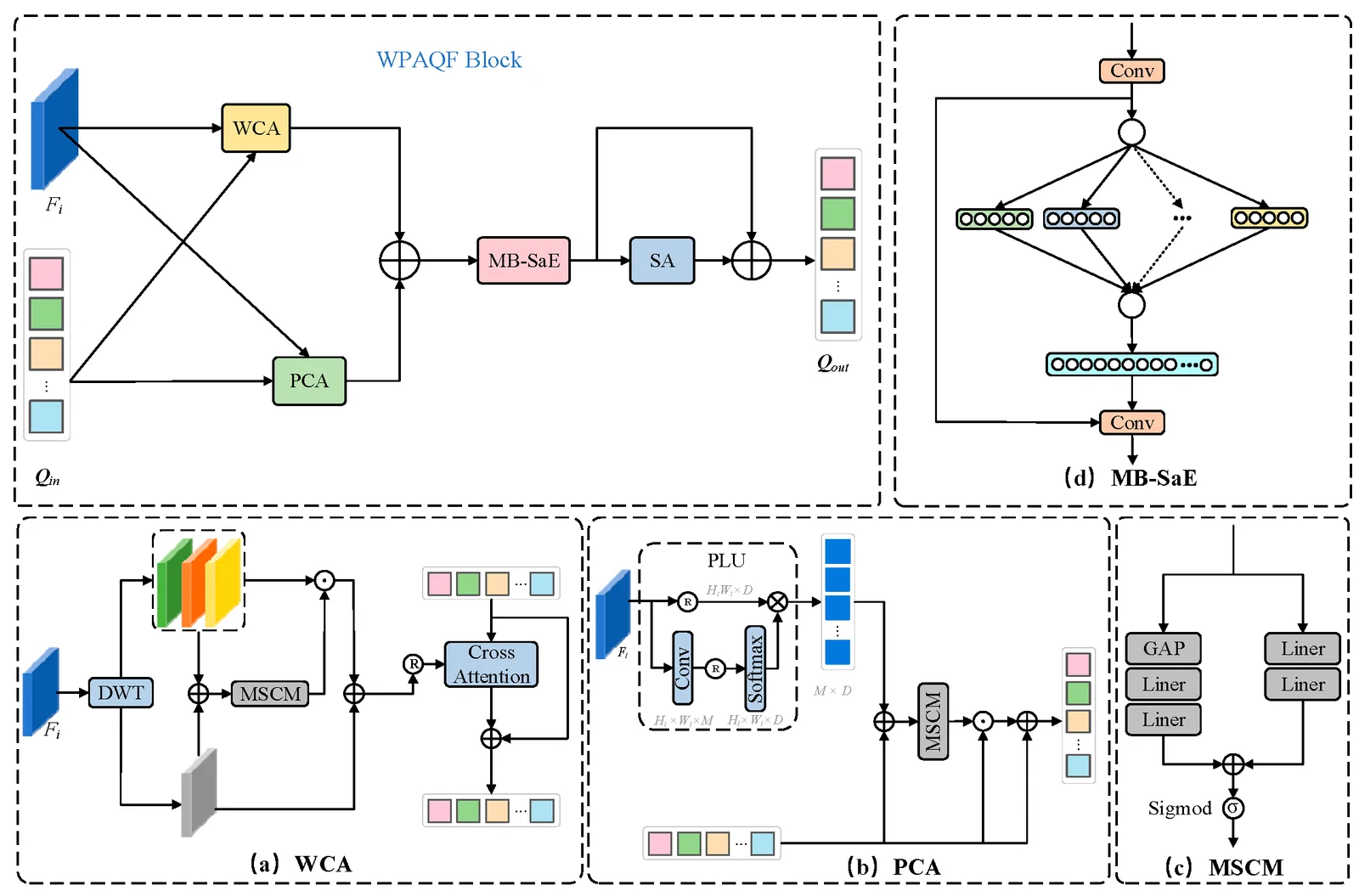
Architecture of WPAQF. (**a**) WCA updates queries using wavelet-domain information; (**b**) PCA updates queries using spatial semantic prototypes; (**c**) MSCM performs intra-branch multi-scale channel modulation; (**d**) MB-SaE adaptively recalibrates the two query branches before query self-attention.

Due to the uneven staining, background tissue texture and scanning noise in glomerular tissue images, the high-frequency response may contain some invalid information. Therefore, WCA dynamically filters different frequency responses by global- versus local-modulation weights, enabling the model to enhance boundary information with structural significance while suppressing interference caused by staining changes and non-target textures. The Multi-Scale Channel Modulation Module (MSCM) in Figure 5 is located inside the WCA and is used to further optimize the channel response in the frequency domain branch and improve the selection ability of effective features.

(2) Prototype-guided cross-attention (PCA)

Since the number of spatial tokens still contains a large amount of background redundant information after multiple downsampling of deep features, direct pixel-level interaction is prone to interference from non-target regions. In order to enhance the semantic consistency within the glomerular region, PCA was introduced into the model, and the spatial features were aggregated into a small number of representative semantic prototypes through the pixel-prototype soft allocation mechanism. Equation (17) represents the fact that WCA updates the shared query using frequency-domain enhanced features. Equation (18) represents the fact that pixel features are aggregated into semantic prototypes according to soft assignment weights. Equation (19) represents the fact that PCA updates the shared query with the prototype matrix as key and value. A represents the pixel-prototype soft assignment matrix, Flatten represents spatial expansion, Attn and Norm represent cross-attention and layer normalization, respectively, and two Q with different superscripts represent queries after WCA and PCA updates, respectively.

(17) $$Q_{i}^{w}=Norm\left[Q_{i}+Attn\left(Q_{i},E_{i}^{fre},E_{i}^{fre}\right)\right]$$

(18) $$P_{i}=A_{i}^{⊤}\;Flatten\left(E_{i}\right)$$

(19) $$Q_{i}^{p}=Norm\left[Q_{i}+Attn\left(Q_{i},P_{i},P_{i}\right)\right]$$

WCA mainly focuses on boundary and texture compensation in frequency space, so that fine-grained information such as glomerular capsule wall and capillary structure can be obtained. PCA reduces the influence of background regions through prototype aggregation, so that regions with similar pathological semantics can form a more stable representation. The two methods enhance the deep-feature expression from the aspects of detail recovery and semantic organization, respectively, and provide more discriminative jump information for the subsequent decoding stage.

(3) Dual-way query Adaptive Fusion (MB-SaE)

After dimensional alignment, the WCA and PCA query outputs are combined by element-wise addition, as shown in Equation (20). MB-SaE then generates channel weights from the fused query representation, as defined in Equation (21). The recalibrated representation is subsequently processed by query self-attention with a residual update, as shown in Equation (22). Here, MB-SaE denotes the multi-branch squeeze-and-excitation module, and SA denotes query self-attention.

(20) $$Q_{i}^{f}=Q_{i}^{w}+Q_{i}^{p}$$

(21) $$e_{i}=MB-SaE\left(Q_{i}^{f}\right)$$

(22) $$Q_{i}^{out}=Norm\left\{Q_{i}^{f}⊙e_{i}+SA\left(Q_{i}^{f}⊙e_{i}\right)\right\}$$

MSCM modulates representations within the WCA and PCA pathways, whereas MB-SaE acts on their summed query output. These operations, therefore, occupy different positions in the module. As formulated in Equations (20)–(22), MB-SaE performs channel recalibration of the fused representation; it does not assign separate explicit weights to the WCA and PCA outputs.

After rearrangement, projection, and scale recovery, the fused query is transformed into spatial features that match the corresponding decoding stage, which are input to the decoder through E4 and E5 jump paths, respectively. WPAQF does not change the feature propagation process in KAN coding path, but, at the stage when deep features have high semantic information density, it recovers local structure information through frequency decomposition, enhances regional semantic expression through prototype aggregation, and uses query interaction to achieve adaptive fusion of the two types of information. Thus, the loss of fine-grained structure and weak boundary information during continuous downsampling is alleviated. The specific structure of WPAQF and its WCA, PCA, MSCM and MB-SaE submodules is shown in Figure 5.

### 2.4. Rule-Based Conversion of Segmentation Outputs into Case-Level MEST-C-Related Indicators

The clinical grading criteria were based on the Oxford Classification and its 2016 update [2,3]. All operational decision rules used to convert segmentation outputs into case-level indicators were fixed before evaluation of the final 40-case cohort and remained unchanged throughout the evaluation. Neither the cases nor their reference labels were used to select, optimize, or recalibrate any decision threshold.

## 3. Results

Based on the patient-level isolation data partitioning, the segmentation accuracy, cross-dataset generalization ability, module contribution and computational complexity of DU-KAN were evaluated on the pediatric IgAN PAS pathological dataset and Synapse public dataset, respectively. Furthermore, the continuous analysis process from pixel-level multi-structure segmentation, pathological element quantification, to case-level rule aggregation was verified by combining qualitative results, class-level indicators and Oxford MEST-C structural decision of 40 independent cases.

### 3.1. Segmentation Performance and Cross-Task Architectural Evaluation

#### 3.1.1. Segmentation Performance on the Pediatric IgAN Pathological Dataset

DU-KAN [9] was compared with U-Net [5], UNet++ [28], ResUNet [29], MK-UNet [24], FEWTD [30], MedDINOv3 [31] and U-KAN on the PAS pathological dataset of children IgAN. To ensure fairness of comparison, all models used the same input size, category settings, patient-level data partitioning, and training process. FEWTD and MedDINOv3 are locally reproduced according to their original decoder structure, visual backbone network and pre-training strategy, respectively.

**Figure 6:**
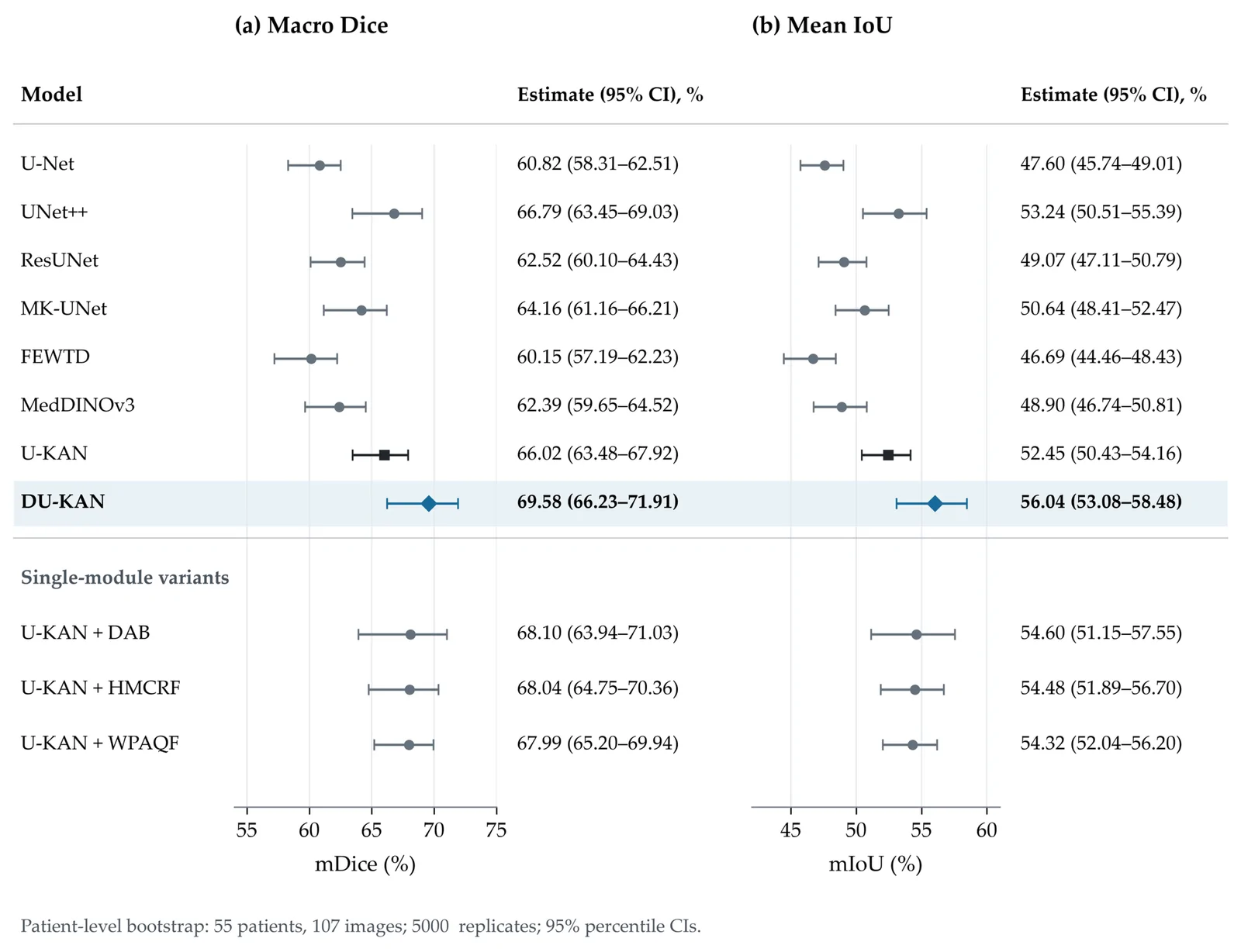
Segmentation performance with patient-level bootstrap confidence intervals. Panels show (**a**) macro-averaged Dice and (**b**) mean IoU across eight classes, including background. Points indicate estimates and horizontal bars indicate 95% percentile confidence intervals from 5000 shared bootstrap resamples of 55 patients contributing 107 images. Blue diamonds identify DU-KAN and dark squares identify U-KAN; the lower rows show single-module variants. Overlap between separate model intervals does not replace paired inference on performance differences.

Table 1 reports overall segmentation performance with patient-level bootstrap confidence intervals. DU-KAN had the highest point estimates among the compared models for the five overall metrics. Figure 6 visualizes the mean Dice and mean IoU estimates for the comparison models and single-module variants.

DU-KAN achieved a mean Dice of 69.58% (95% CI, 66.23–71.91) and a mean IoU of 56.04% (95% CI, 53.08–58.48). Relative to U-KAN, the paired improvements were 3.56 percentage points in mean Dice (95% CI, 1.90–5.26) and 3.59 percentage points in mean IoU (95% CI, 1.83–5.45). The mean Recall difference was 1.51 percentage points (95% CI, −0.19–3.22), indicating uncertainty about the Recall improvement despite its higher point estimate.

Foreground mean Dice, excluding background, was 65.42% (95% CI, 61.61–68.07), with a paired improvement of 4.00 percentage points over U-KAN (95% CI, 2.12–5.96). These results indicate that the observed overlap gains extended to the foreground pathological structures.

#### 3.1.2. Cross-Task Architectural Evaluation on the Synapse Public Dataset

To examine whether the architectural improvements of DU-KAN remain effective in a substantially different medical image modality and segmentation task, comparative experiments were conducted on the Synapse abdominal CT dataset. Consistent data partitioning, input size, and training strategies were used for all models, and the experimental results are shown in Table 2. DU-KAN achieved the best performance across all five overall evaluation indicators, with aAcc, mIoUall, mPrecision, mRecall, and mDiceall of 99.00%, 80.69%, 92.58%, 85.39%, and 88.58%, respectively, showing favorable performance in multi-organ localization, category discrimination, and overall segmentation consistency.

Compared with the basic model U-KAN, the mIoU_all_, mDice_all_ and mRecall of DU-KAN were increased by 8.57, 6.34 and 7.27 percentage points, respectively, indicating that the model could effectively improve the spatial overlap ability of organ regions and the capture ability of target regions. At the same time, the mPrecision was increased by 4.22 percentage points, indicating that DU-KAN did not introduce more false segmentation regions while improving the Recall rate, and achieved a good balance between Precision and integrity.

After further excluding the background category, the average Dice score of DU-KAN on the eight foreground organs reached 87.21%, which was 7.10 percentage points higher than that of U-KAN, indicating that the improvement of DU-KAN’s performance was mainly due to the enhancement of the representation ability of the foreground organ structure, rather than the contribution of the background region. Taken together, these results show that the performance gains of DU-KAN relative to U-KAN are retained in a substantially different segmentation setting, providing complementary evidence for the cross-task generalizability of the proposed architectural design.

### 3.2. Ablation Experiments and Complexity Analysis

In order to verify the effectiveness of the three modules, DAB, HMCRF and WPAQF, a single module was introduced into the ablation experiment with U-KAN as the baseline, and the performance improvement of the three combined configurations was further evaluated. All experiments used the same data partitioning, training strategy, and evaluation metrics, and the results are shown in Table 3.

In the pathological-image segmentation task of children IgAN, mDice_all_ increased by 2.08, 2.02 and 1.97 percentage points, and mIoU_all_ increased by 2.15, 2.04 and 1.87 percentage points, respectively, after introducing DAB, HMCRF and WPAQF alone. All the three modules can effectively enhance the feature-representation ability of pathological regions. Among them, DAB achieved slightly better single-module performance, indicating its stronger adaptability in complex-lesion boundaries and fine-grained structure segmentation. After further fusion of three modules, the mDice_all_ and mIoU_all_ of DU-KAN reached 69.58% and 56.04%, which was still 1.48 and 1.44 percentage points higher than the best single-module configuration, which verified that multi-module collaboration could further improve the accuracy of pathological-structure segmentation.

In the Synapse abdominal CT multi-organ segmentation task, each module also showed stable gains, among which WPAQF achieved the most obvious improvement; mDice_all_ and mIoU_all_ increased by 3.59 and 4.87 percentage points, respectively, indicating that it had more advantages in organ region modeling and spatial information capture. Complete DU-KAN further improved mDice_all_ and mIoU_all_ to 88.58% and 80.69%, respectively, which was still 2.75 and 3.70 percentage points higher than the optimal single-module configuration. The average Dice of foreground organs after removing background also showed the same trend, indicating that the performance improvement of DU-KAN mainly came from the effective segmentation of the real target region, rather than the background-category contribution.

Across the two datasets, the evaluated module configurations improved segmentation performance relative to U-KAN, and the complete model achieved higher performance than the individual configurations. These results support the effectiveness of the combined architecture under the evaluated settings. However, the module-level comparisons do not isolate the effects of delayed fusion, stage-specific placement, or individual operations within HMCRF and WPAQF.

In addition to segmentation accuracy, the computational cost of each configuration was evaluated in terms of parameter counts, FLOPs, and inference time (Table 4). The results show that different modules introduce different levels of computational overhead. DAB increases only 0.137 M parameters and 0.306 G FLOPs compared with U-KAN, indicating a relatively small computational overhead. HMCRF introduces a moderate increase in complexity due to multi-scale feature fusion, whereas WPAQF contributes the largest computational overhead because of additional wavelet decomposition and attention-based feature refinement. After integrating all modules, DU-KAN requires 61.417 M parameters and 14.352 G FLOPs, with an inference time of 1.44 ms.

Compared with U-KAN, DU-KAN improves the mean Dice score by 3.56 percentage points, while increasing the parameter count from 25.360 M to 61.417 M, FLOPs from 6.903 G to 14.352 G, and inference time from 0.69 ms to 1.44 ms. Thus, the segmentation improvement is accompanied by increased computational and inference costs. This trade-off may be acceptable in accuracy-oriented pathological-image analysis where segmentation performance is prioritized, but it may limit deployment in highly resource-constrained or latency-sensitive settings.

Furthermore, Table 5 places the computational requirements of DU-KAN in the context of existing segmentation networks. DU-KAN is computationally more demanding than lightweight CNN-based models such as MK-UNet and U-Net, as well as the baseline U-KAN. However, its FLOPs and inference time remain substantially lower than those of several higher-complexity architectures, including UNet++, MedT, ResUNet, and ResUNet++. Therefore, DU-KAN should not be regarded as a lightweight architecture; rather, it represents a trade-off in which improved segmentation performance is accompanied by increased computational cost. This characteristic makes the model more appropriate for scenarios where segmentation accuracy is prioritized over strict computational or latency constraints.

### 3.3. Qualitative Performance and Category-Level Validation

#### 3.3.1. Qualitative Results and Sample-Difficulty Analysis

**Figure 7:**
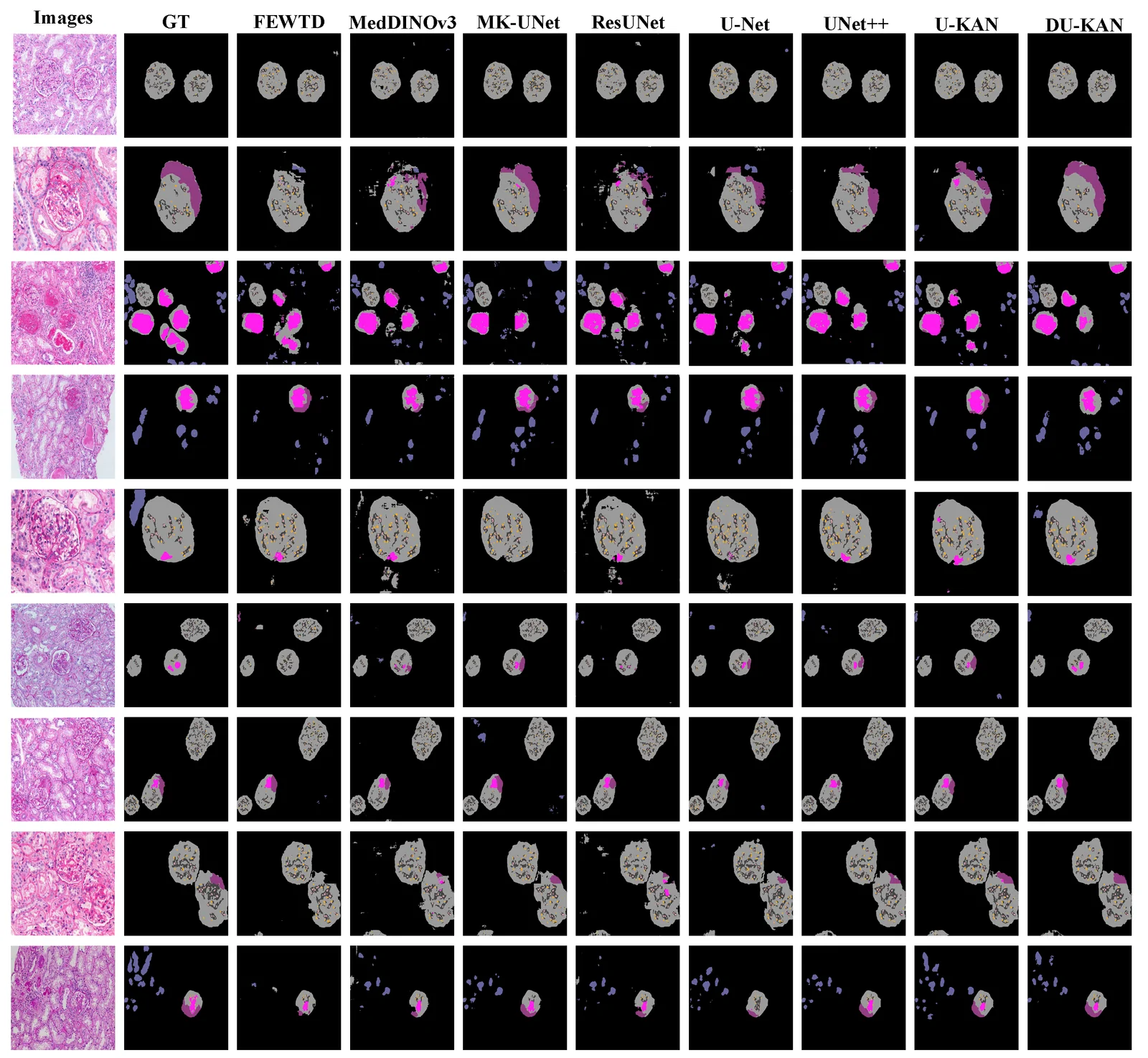
Overall qualitative comparison of segmentation methods on the in-house pediatric IgAN pathology dataset.

**Figure 8:**
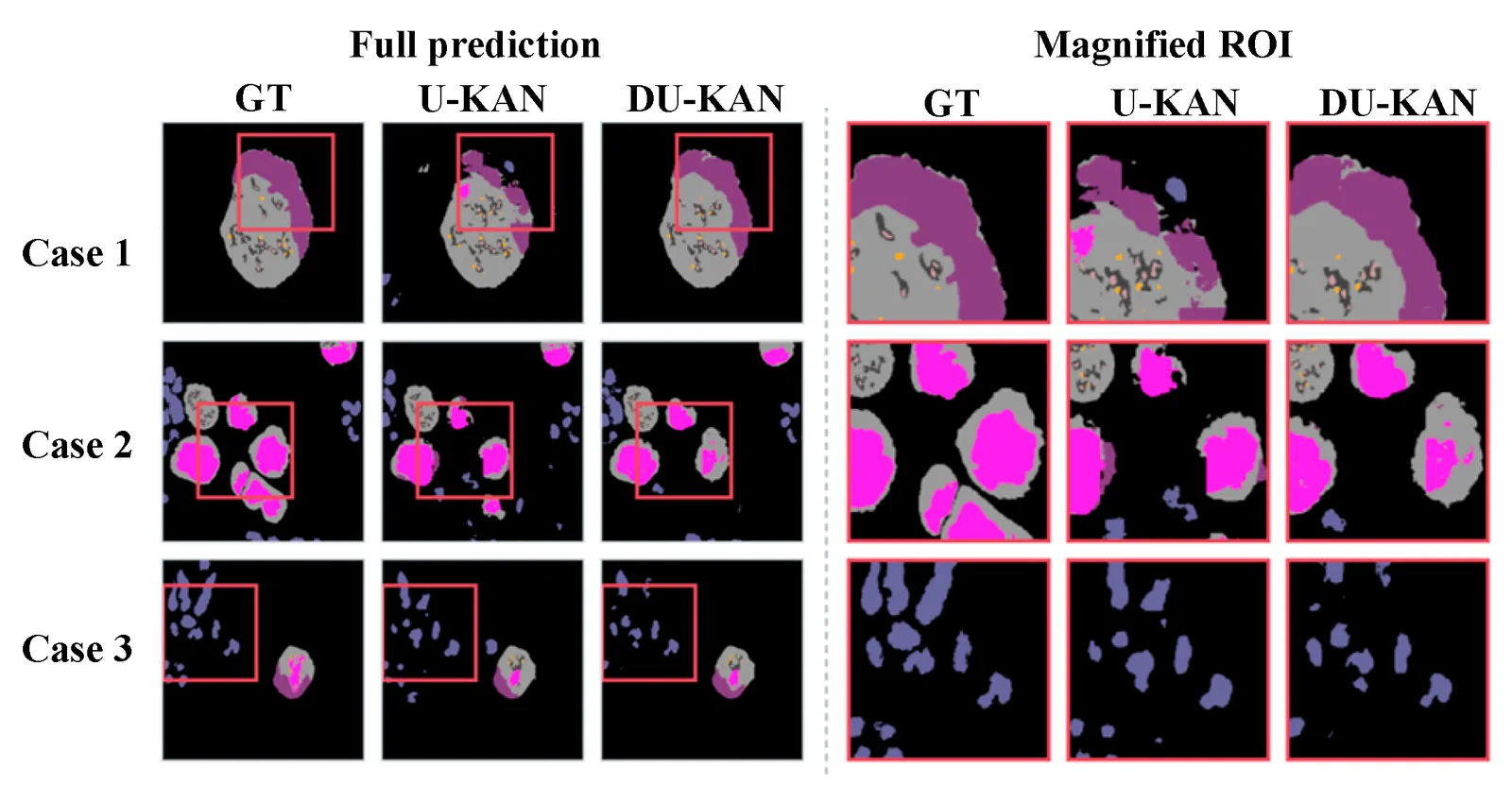
Local comparison of shared regions in representative difficult cases.

In order to visually analyze the segmentation differences of different models on complex pathological structures, Figure 7 shows nine sets of test samples and their prediction results, focusing on the comparison of the performance of each model in lesion area integrity, boundary recovery, small target recognition and background false-positive suppression. Figure 8 further selected typical difficult samples with irregular boundaries, coexistence of multiple pathological structures, and dense fine-grained targets, and locally magnified the same regions of interest in GT, U-KAN, and DU-KAN prediction results to observe the segmentation differences of the model on complex boundaries and tiny structures. It should be noted that the visualization results are mainly used to assist in explaining the prediction characteristics of the model, and the model performance is still mainly judged by the quantitative evaluation results on the complete test set.

From the overall comparison results, DU-KAN could cover the lesion area more completely in most display samples, and the predicted contour had high consistency with GT. In contrast, some contrast models still have problems such as local missing of the lesion area, expansion of the prediction boundary, fracture of the target structure, and isolated false detection of the background area. The differences between the models are even more pronounced for samples where large irregular lesions coexist with discrete tiny targets. DU-KAN can not only maintain the integrity of the main lesion region, but also show better modeling ability for the spatial relationship between pathological structures in different locations, thus reducing local missed segmentation and false response of non-target regions.

The local magnification results shown in Figure 8 further present the prediction differences between different models in difficult regions. In Case 1 and Case 2, U-KAN still showed local boundary expansion, target fracture, and adhesion of adjacent structures, while DU-KAN showed a more concentrated predicted response, the lesion contour was closer to GT, and the edge continuity was improved. In Case 3, both U-KAN and DU-KAN omitted some discrete micro structures to varying degrees, indicating that targets with low pixel proportion, irregular morphology and weak contrast with surrounding tissue are still the main error sources of the current model.

These visual differences are generally consistent with the design goals of DAB, to enhance contextual information across regions, those of HMCRF, to adapt pathological structures at different scales, and those of WPAQF, to supplement boundary details in deep features. However, the visualization results cannot distinguish the actual contributions of each module alone, and the relevant performance gains still need to be judged in combination with module ablation experiments.

Although DU-KAN improved the lesion region integrity and boundary continuity in most samples, a certain degree of missed segmentation and contour deviation were still observed for very small targets and local weak-boundary regions, indicating that the model still has room for further improvement in small-target Recall and fine-boundary recovery.

Overall, the visualization phenomena in Figure 7 and Figure 8 are in good agreement with the category-level quantitative results. Compared with U-KAN, the Dice of DU-KAN in tubular atrophy, crescent, endothelial cell and mesangial cell categories increased by 11.55, 4.22, 3.55 and 3.06 percentage points, respectively. The performance improvement was mainly concentrated in the foreground categories with weak boundary, obvious morphological scale change, or low pixel proportion, indicating that the gain of the model did not mainly come from the prediction of background categories, but from the recognition of complex pathological structures and the contour recovery ability. Qualitative results, category-level indicators and ablation experiments complement each other from different aspects, and together illustrate the performance advantages and limitations of DU-KAN in the multi-category segmentation task of pathological images of children with IgAN.

#### 3.3.2. Class-Level Verification of Pathological Structure of IgAN in Children

**Figure 9:**
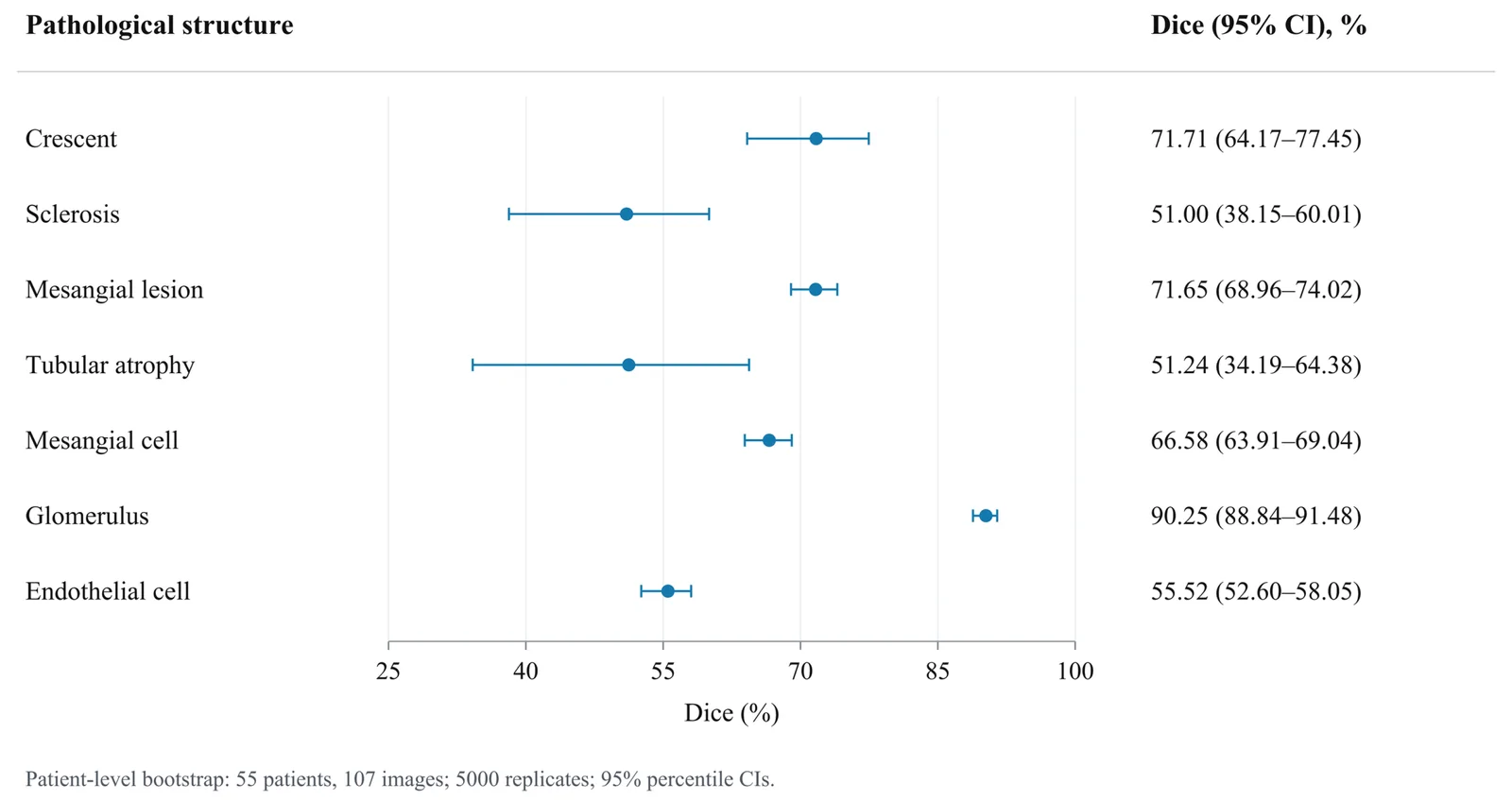
Class-specific Dice scores of DU-KAN. Points indicate estimates and horizontal bars indicate 95% percentile confidence intervals from 5000 patient-level cluster bootstrap resamples of 55 patients contributing 107 images. All images from each selected patient were retained together, with multiplicity for repeatedly selected patients. The seven foreground classes are shown; background is excluded.

Class-specific Dice scores and paired differences between U-KAN and DU-KAN are reported with patient-level bootstrap confidence intervals in Table 6. Figure 9 shows the Dice estimates and confidence intervals for the seven foreground classes.

DU-KAN had higher Dice point estimates than U-KAN across all foreground classes. However, the Precision of these estimates and the evidence for improvement varied by class.

DU-KAN achieved Dice scores of 51.00% (95% CI, 38.15–60.01) for sclerosis and 51.24% (95% CI, 34.19–64.38) for tubular atrophy, indicating substantial uncertainty in these estimates. The paired improvement in tubular atrophy Dice was 11.55 percentage points (95% CI, 6.23–17.24). Although the Dice point estimates also increased for crescents and sclerosis, their paired difference intervals included zero: 4.22 percentage points (95% CI, −2.71–11.79) and 1.92 percentage points (95% CI, −4.20–7.99), respectively.

The paired Dice difference intervals for mesangial lesions, mesangial cells, glomeruli, and endothelial cells did not include zero. These class-specific comparisons were exploratory, and were not adjusted for multiple comparisons; higher point estimates should therefore not be interpreted as uniform evidence of improvement across all structures.

Table 7 further compares the contribution of each module to different pathology categories. The single-module experiment showed that DAB improved crescent, mesangial lesion and tubular atrophy more prominently. HMCRF showed greater gains in tubular atrophy, crescent and endothelial cells. WPAQF showed a strong improvement effect on tubular atrophy, sclerosis and crescent. All three modules improved the overall segmentation performance, but the mDice_all_ of the complete DU-KAN reached 69.58%, which was 1.48, 1.54 and 1.59 percentage points higher than the three single-module configurations, respectively, and achieved the highest Dice in all categories except sclerosis.

The above results indicate that DAB, HMCRF and WPAQF do not simply produce repetition gain, but improve shallow local representation, cross-layer heterogeneous feature fusion and deep semantic compensation, respectively. When the three methods are combined, DU-KAN can take into account the large-scale structure, small-lesion area and fuzzy boundary more fully, so as to obtain a more stable and balanced class-level segmentation performance. It should be pointed out that crescent and sclerosis still have the phenomenon of improved Precision and decreased Recall, indicating that there is still room for further optimization of the model between suppressing false detection and maintaining Recall of small lesions.

#### 3.3.3. Class-Level Cross-Task Evaluation on the Synapse Dataset

To further examine the transferability of the architectural improvements across different imaging modalities and segmentation targets, a class-level evaluation was conducted on the Synapse abdominal CT multi-organ segmentation dataset. Table 8 reports the Dice scores for the eight foreground organs, with their arithmetic mean denoted as mDiceorgan, which differs from mDiceall by excluding the background category. DU-KAN achieved an mDiceorgan of 87.21%, 7.10 percentage points higher than U-KAN, indicating that the performance gains associated with the proposed architecture were retained when transferred from pediatric IgAN pathology segmentation to CT multi-organ segmentation.

In terms of category results, DU-KAN improved the performance of all organ categories, especially for Stomach, Pancreas and Gallbladder, which were difficult to segment, with Dice increased by 16.37, 12.54 and 12.41 percentage points, respectively. The above organs usually have the characteristics of large morphological changes, fuzzy boundaries and small target regions. DU-KAN can improve Precision and Recall at the same time, indicating that DU-KAN can effectively alleviate the problem of missed detection while reducing false detection, and enhance the localization and segmentation ability of complex organ regions.

For organs with high baseline performance, such as Aorta, Kidney_L, Kidney_R, Liver and Spleen, DU-KAN still achieved 3.26, 1.90, 4.12, 2.70 and 3.44 percentage points, respectively. In conclusion, DU-KAN can not only significantly improve the segmentation results of difficult organs, but also further optimize the high-baseline categories, maintaining stable gains in organ categories with different sizes, shapes and contrast, showing consistent performance gains across organs with different sizes, shapes, and contrast characteristics.

Table 9 further compares the differences in the roles of different modules on each organ category. According to mDice_organ_, the performance of the model was improved from 80.11% to 82.32%, 83.21% and 84.13% when DAB, HMCRF and WPAQF were introduced separately, indicating that all three modules could improve the segmentation ability of the model on the public datasets. Among them, WPAQF achieved the largest single-module average gain, and the improvement of Stomach, Gallbladder, Kidney_R and Liver was more obvious. HMCRF showed better performance in complex organs such as Pancreas, Gallbladder and Stomach. DAB achieved higher Dice on Kidney_L, Kidney_R, Aorta and Stomach, which reflected that the enhancement effects of different modules on organ features were complementary.

The mDice_organ_ of intact DU-KAN reached 87.21%, which was 4.89, 4.00 and 3.08 percentage points higher than that of U-KAN-DAB, U-KAN-HMCRF and U-KAN-WPAQF, respectively, and the highest Dice was obtained in the other seven organs except Kidney_L. In the difficulty categories of Gallbladder, Pancreas and Stomach, especially, the full model improved by 10.23, 6.65 and 2.34 percentage points, respectively, compared with the optimal single-module configuration, indicating that the multi-module collaboration could further make up for the limitations of the single module in complex boundaries and low-contrast regions. In summary, DU-KAN achieves consistent improvement in all organ categories on the public Synapse dataset, and maintains stable advantages in difficult organs and high-baseline organs. In summary, DU-KAN achieved consistent improvements across organ categories on the public Synapse dataset and maintained gains in both difficult organs and high-baseline organs. These findings provide additional class-level evidence that the architectural improvements of DU-KAN yield transferable segmentation benefits in a substantially different task, supporting cross-task architectural generalization rather than disease-specific external validation.

### 3.4. Oxford MEST-C Case-Level Structured Decision Results

Based on the results of multi-structure segmentation, the connectivity domain of mesangial cells, endothelial cell burden, sclerosis foci, crescent and extraocular atrophy proportion were aggregated into case-level indicators, and Oxford MEST-C structured decision was generated, according to prespecified rules. The independent case cohort consisted of 40 cases and 75 PAS images, and the artificial truth values were given by the clinical pathologist. This cohort evaluated the discriminative power and result traceability of the “segmentation–quantification–aggregation” process at the case level, and, together with the main segmentation experiment, formed a multi-level verification from pixel-level performance to case-level pathological interpretation.

#### 3.4.1. Example of Structured Decision Output

Table 10 lists the judgment basis, representative structured data and output results of items M, E, S, T and C, which are used to show how the pixel-level segmentation results are transformed into case-level pathological evidence that can be reviewed. Examples output M1, E1, S1, T1, and C1, respectively. M and E reserved the cell count or area burden, S and C reserved the presence of lesions and the information of the glomerulus to which they belonged, and T reserved the area of atrophy and the area of effective tissue outside the glomerulus. Thus, case-level decision can be traced back to the corresponding structural indicators, rather than the direct output of uninterpretable classification conclusions from the image.

#### 3.4.2. Judging Performance of Single Item and Combination

In order to present the independent discrimination performance of each pathological dimension and the combined determination results of MEST-C at the same time, Table 11 summarizes the consistency rate, sensitivity, specificity and continuous-scoring performance of M, E, S, T and C. The consistency rate of the five single indicators was more than 84%. Among them, the M, E, S, T, and C of 26 cases were completely consistent with the manual results, and the overall consistency rate was 65.00%.

Table 11 shows that the performance of S term is relatively stable in the identification of positive lesions. The overall agreement rate of item E was 87.50%, but the sensitivity was 37.50%, indicating that low-burden endothelial hyperplasia remained a major source of missed detection. The agreement rate of T was 97.50%, with a sensitivity of 50.00% and AP of 0.53; therefore, the high agreement rate should be interpreted together with the class distribution and threshold-related discrimination performance. The simultaneous false-positive and false-negative results of item C suggest that the presence of OR aggregation is helpful to cover the focal lesions, but its specificity is still affected by the image-level error.

#### 3.4.3. Confusion Features and Threshold Discrimination Ability

**Figure 10:**
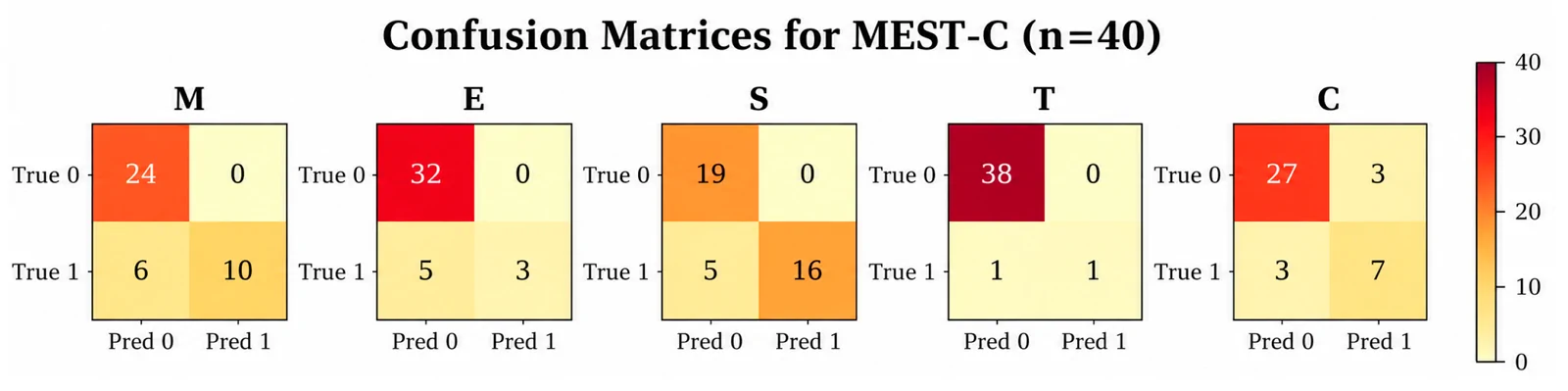
Confusion matrices for structured M, E, S, T, and C determination in 40 cases.

The case-level confusion matrix is shown in Figure 10. There were no false-positive cases in terms M, E, S and T, and the errors were 6, 5, 5 and 1 false-negative cases, respectively, indicating that the current rule was generally conservative. Term C, with three false positives and three false negatives, was the most complex indicator of the direction of error.

**Figure 11:**
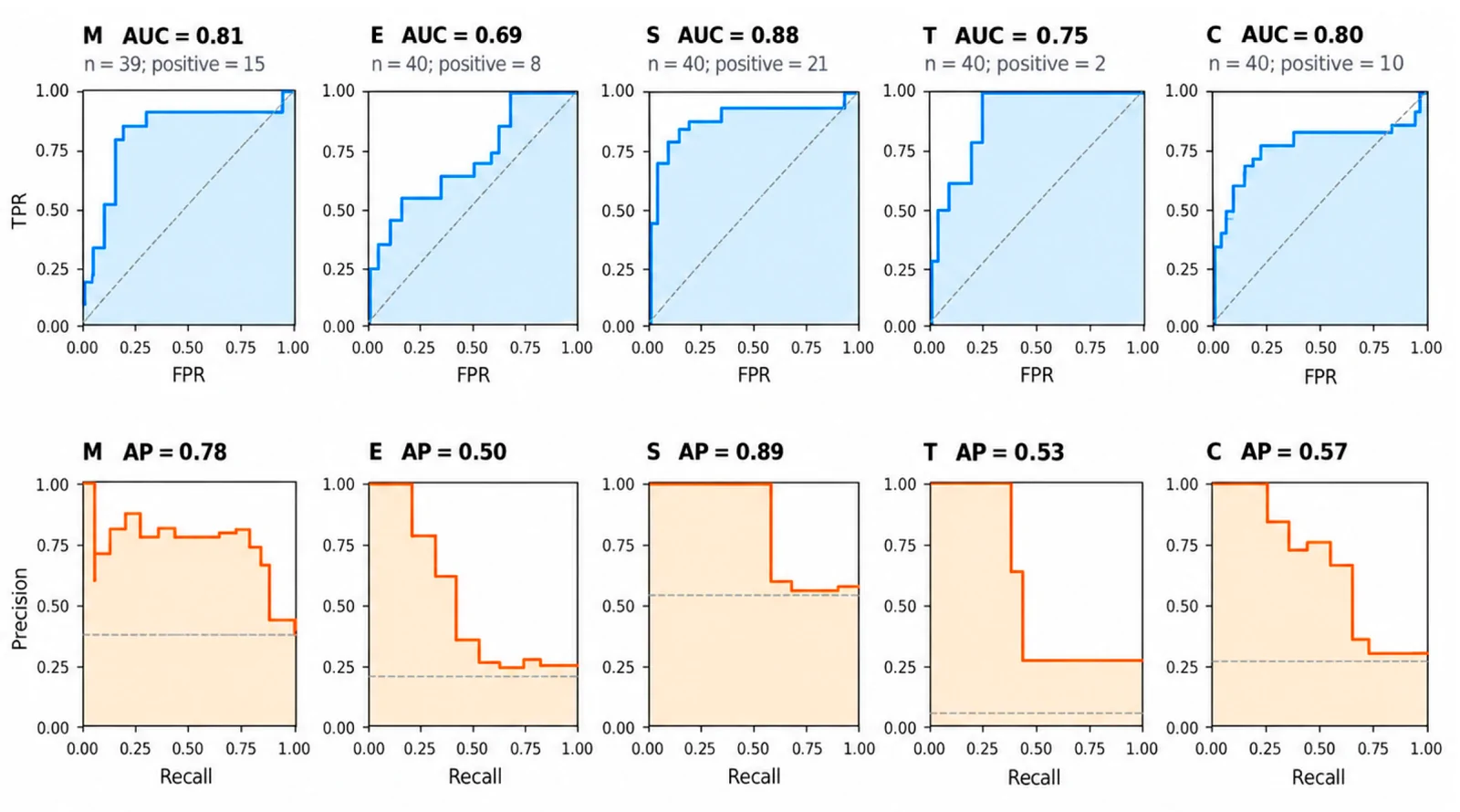
ROC and Precision–Recall curves for the five case-level structured indicators M, E, S, T, and C.

Furthermore, ROC and Precision–Recall curves were plotted based on proportion of high mesangial burden, mean endothelial burden, pixel volume of sclerosis foci, proportion of extraocular atrophy, and crescent aggregation score (Figure 11). The S, C and M terms showed good ranking ability, while the E term showed relatively limited discrimination for low-burden lesions. T ROC-AUC was 0.75 and AP was 0.53, reflecting the difference between ROC and PR under the condition of few positive cases. Combined with the positive proportion and score definition of each index, ROC and PR curves could describe the threshold discrimination characteristics of continuous indicators at case level, from different perspectives.

## 4. Discussion

### 4.1. Main Findings and Research Contributions

DU-KAN improved internal multi-structure segmentation in pediatric IgAN PAS images, achieving an eight-class mean Dice of 69.58% (95% patient-level bootstrap CI, 66.23–71.91). The paired improvement over U-KAN was 3.56 percentage points (95% CI, 1.90–5.26); foreground mean Dice was 65.42%, with an improvement of 4.00 percentage points (95% CI, 2.12–5.96). The principal methodological contribution is the stage-specific architectural design: DAB introduces parallel convolutional and MaxViT pathways in the shallow encoder, while HMCRF and WPAQF adapt established mechanisms for shallow-feature fusion and deep-skip enhancement. The contribution, therefore, concerns architectural design and task-specific integration, rather than new computational primitives. Segmentation outputs were additionally converted into reviewable MEST-C-related indicators, although improved segmentation did not establish reliable case-level assessment.

### 4.2. Interpretation of Results and Comparison with Previous Studies

The ablation results support the combined architecture: DAB, HMCRF, and WPAQF individually increased mean Dice by 2.08, 2.02, and 1.97 percentage points over U-KAN, respectively, and the full model achieved the highest overall score. However, these comparisons do not isolate the effects of stage placement, delayed fusion, or individual operations within the adapted modules. The proposed explanations involving contextual modeling, feature recalibration, and boundary preservation therefore remain interpretations, rather than direct evidence of internal mechanisms.

Residual segmentation errors remain relevant to case-level assessment. Dice scores were only 51.00% for sclerosis, 51.24% for tubular atrophy, and 55.52% for endothelial cells. Moreover, the exploratory paired-confidence intervals for crescent and sclerosis improvements included zero. Missed sclerosis or crescent lesions can change presence-based decisions, even when boundary overlap improves. Endothelial under-segmentation can lower the burden measure used for E, while errors in atrophic area or the reference tissue denominator can alter T estimates near grading thresholds. M and proportion-based C grading are additionally vulnerable to glomerular counting and lesion-attribution errors. Thus, higher mean Dice does not necessarily translate into more reliable MEST-C-related decisions.

This distinction was evident in the exploratory 40-case evaluation. Complete agreement across the five assessed indicators was 65.00% (26/40), with sensitivities of 37.50% for E (3/8) and 50.00% for T (1/2). The 97.50% agreement for T was dominated by 38 negative cases; its sensitivity and AP of 0.53 were based on only two positive cases, and cannot establish reliable detection. The absence of false positives for M, E, S, and T in this small cohort should, likewise, not be interpreted as established specificity. These findings support further investigation of structured evidence generation, while clinical utility remains unproven.

Previous renal segmentation and MEST-C studies have already demonstrated multi-structure quantification and segmentation-based scoring [11,12,13,14,15,16]. DU-KAN extends this direction through a stage-adapted architecture and explicit case-level aggregation, but superiority over existing clinical scoring workflows was not evaluated. Synapse organ mean Dice of 87.21% provides complementary evidence of cross-task architectural generalization. Because Synapse involves abdominal CT rather than renal histopathology, it does not establish external validity in pediatric IgAN.

The segmentation gain also carries substantial computational cost. Compared with U-KAN, parameters increased from 25.360 M to 61.417 M and FLOPs from 6.903 G to 14.352 G, approximately 2.42-fold and 2.08-fold increases for a 3.56-percentage-point mean Dice improvement. DU-KAN should, therefore, not be considered lightweight. The inference times reported in Table 4 do not establish end-to-end biopsy processing speed, which also depends on image coverage, sliding-window inference, preprocessing, and case-level aggregation. Whether or not the accuracy gain justifies the additional resources requires evaluation in the intended deployment workflow.

### 4.3. Study Limitations

This study has several limitations. The segmentation dataset comprised 532 selected PAS images from 278 children at one center, with only 55 patients in the held-out test set. Patient-level separation reduces leakage, and cluster bootstrapping quantifies sampling uncertainty, but neither establishes performance across institutions. Because predictions were fixed during bootstrapping, the confidence intervals do not capture variability from retraining or alternative data splits.

The 40-case assessment contained few positive cases for some indicators, particularly T. Although operational rules were fixed before evaluation, robustness to their threshold choices has not been demonstrated. The evaluated outputs should also be distinguished from complete Oxford grading: C was assessed as a binary presence indicator, and T segmentation targeted atrophy-related regions, rather than separately validating interstitial fibrosis. These choices limit interpretation of agreement as full MEST-C grading accuracy.

Selected high-power fields may not adequately represent biopsy-wide lesion burden or cortical involvement. Semantic segmentation also lacks explicit instance-level lesion-to-glomerulus modeling, introducing uncertainty in attribution and aggregation. Before clinical use, the complete workflow requires independent multicenter validation across staining batches and scanners, adequate positive-case representation, whole-slide assessment, and direct comparison with multiple renal pathologists, using a predefined reference standard.

## 5. Conclusions

DU-KAN improved internal multi-structure segmentation in pediatric IgAN PAS images, with a mean Dice gain of 3.56 percentage points over U-KAN (95% CI, 1.90–5.26), at increased computational cost. Synapse results support cross-task architectural generalization, not external validation in pediatric IgAN. The framework also enabled segmentation-derived, reviewable MEST-C-related quantification.

Clinical reliability remains unestablished. In the exploratory 40-case cohort, complete five-indicator agreement was 65.00%, while sensitivity was 37.50% for E and 50.00% for T. T agreement of 97.50% largely reflected negative cases; only two cases were positive, and AP was 0.53. These results support further methodological investigation, but do not establish readiness for clinical use. Independent multicenter validation of the complete workflow, including threshold robustness, whole-slide assessment, adequate positive-case representation, and comparison with multiple renal pathologists, is required before clinical implementation.

## Figures and Tables

**Table 1:** Quantitative comparison of the pediatric IgAN pathology dataset: estimates and 95% confidence intervals (%).

Model	aAcc	mIoU_all_	mPrecision	mRecall	mDice_all_
U-Net	93.85 (92.59–94.96)	47.60 (45.74–49.01)	64.59 (61.61–67.19)	59.46 (57.03–61.48)	60.82 (58.31–62.51)
UNet++	94.86 (93.74–95.82)	53.24 (50.51–55.39)	71.65 (68.24–74.55)	64.25 (60.89–66.95)	66.79 (63.45–69.03)
ResUNet	93.71 (92.34–94.92)	49.07 (47.11–50.79)	67.77 (64.45–70.75)	60.08 (57.68–62.35)	62.52 (60.10–64.43)
MK-UNet	94.14 (93.07–95.10)	50.64 (48.41–52.47)	67.29 (64.31–69.85)	62.04 (58.98–64.62)	64.16 (61.16–66.21)
FEWTD	93.05 (91.70–94.21)	46.69 (44.46–48.43)	61.47 (58.57–64.71)	59.22 (55.97–61.86)	60.15 (57.19–62.23)
MedDINOv3	93.64 (92.29–94.86)	48.90 (46.74–50.81)	67.00 (63.42–69.99)	60.48 (57.80–62.88)	62.39 (59.65–64.52)
U-KAN	94.48 (93.35–95.50)	52.45 (50.43–54.16)	68.05 (65.02–70.67)	64.74 (62.13–67.18)	66.02 (63.48–67.92)
DU-KAN	95.36 (94.34–96.27)	56.04 (53.08–58.48)	75.49 (72.63–77.87)	66.25 (62.92–69.02)	69.58 (66.23–71.91)

Values are point estimates with 95% percentile confidence intervals in parentheses, based on 5000 patient-level bootstrap resamples of 55 patients contributing 107 images. Overall macro averages include eight classes, including background.

**Table 2:** Quantitative comparison on the Synapse dataset (%).

Model	aAcc	mIoU_all_	mPrecision	mRecall	mDice_all_
U-Net	98.26	68.88	84.36	76.50	79.59
UNet++	97.75	61.63	83.26	71.65	70.86
ResUNet	98.04	65.72	80.68	73.20	75.82
MK-UNet	98.12	64.08	82.47	72.13	75.56
FEWTD	97.96	59.67	83.07	65.45	71.25
MedDINOv3	97.97	58.99	88.07	64.50	66.22
U-KAN	98.43	72.12	88.36	78.12	82.24
DU-KAN	99.00	80.69	92.58	85.39	88.58

**Table 3:** Module ablation results on two datasets (%).

Model	IgAN mDice_all_	IgA mIoU_all_	Synapse mDice_all_	Synapse mIoU_all_
Baseline	66.02	52.45	82.24	72.12
+ DAB	68.10	54.60	84.22	75.07
+ HMCRF	68.04	54.48	85.01	75.66
+ WPAQF	67.99	54.32	85.83	76.99
Full model	69.58	56.04	88.58	80.69

**Table 4:** Parameter counts, computational complexity, and inference efficiency of U-KAN configurations.

Model	Params (M)	FLOPs (G)	Inference Time (ms)
U-KAN	25.360	6.903	0.69
U-KAN-DAB	25.497	7.209	0.72
U-KAN-HMCRF	32.464	7.180	0.72
U-KAN-WPAQF	54.022	13.475	1.35
DU-KAN	61.417	14.352	1.44

**Table 5:** Comparison of model complexity and inference efficiency with existing segmentation networks.

Model	Params (M)	FLOPs (G)	Inference Time (ms)
MK-UNet	0.936	1.358	0.14
UNet	1.298	3.695	0.37
UNet++	22.944	88.523	8.85
MedT	32.656	205.248	20.52
ResUNet	32.678	205.248	20.52
ResUNet++	40.632	158.213	15.82
FEWTD	125.611	19.743	1.97
U-KAN	25.360	6.903	0.69
DU-KAN	61.417	14.352	1.44

**Table 6:** Class-wise Dice scores and paired differences with 95% confidence intervals on the pediatric IgAN pathology dataset.

Category	U-KAN Dice (%)	DU-KAN Dice (%)	ΔDice (Percentage Points)
background	98.24 (97.68–98.71)	98.66 (98.18–99.05)	0.42 (0.25–0.62)
crescent	67.49 (61.64–70.64)	71.71 (64.17–77.45)	4.22 (−2.71–11.79)
sclerosis	49.07 (37.79–57.51)	51.00 (38.15–60.01)	1.92 (−4.20–7.99)
mesangial lesion	68.84 (66.08–71.21)	71.65 (68.96–74.02)	2.81 (1.60–4.00)
tubular atrophy	39.69 (25.69–52.54)	51.24 (34.19–64.38)	11.55 (6.23–17.24)
mesangial cell	63.53 (60.65–66.13)	66.58 (63.91–69.04)	3.06 (1.93–4.15)
glomerulus	89.32 (87.89–90.61)	90.25 (88.84–91.48)	0.92 (0.51–1.40)
endothelial cell	51.97 (48.92–54.68)	55.52 (52.60–58.05)	3.55 (2.08–5.08)
mDiceall	66.02 (63.48–67.92)	69.58 (66.23–71.91)	3.56 (1.90–5.26)
Foreground mDice	61.42 (58.52–63.59)	65.42 (61.61–68.07)	4.00 (2.12–5.96)

Values are estimates with 95% patient-level bootstrap confidence intervals in parentheses. ΔDice is DU-KAN minus U-KAN, calculated using identical patient resamples. mDiceall includes background; foreground mDice excludes background. Class-specific intervals are exploratory, and unadjusted for multiple comparisons.

**Table 7:** Class-wise Dice ablation results on the pediatric IgAN pathology dataset (%).

Categories	U-KAN	U-KAN-DAB	U-KAN-HMCRF	U-KAN-WPAQF	DU-KAN
background	98.24	98.53	98.52	98.50	98.66
crescent	67.49	71.52	70.13	69.87	71.71
sclerosis	49.07	48.41	48.47	51.81	51.00
mesangial lesion	68.84	71.02	70.85	69.67	71.65
tubular atrophy	39.69	46.01	46.61	46.97	51.24
mesangial cell	63.53	65.78	65.38	64.36	66.58
glomerulus	89.32	89.64	89.81	89.50	90.25
endothelial cell	51.97	53.91	54.53	53.22	55.52
mDice_all_	66.02	68.10	68.04	67.99	69.58

**Table 8:** Class-wise Dice scores for eight organs on the Synapse dataset (%).

Categories	U-KAN Dice	DU-KAN Dice	ΔDice
Aorta	86.77	90.03	+3.26
Gallbladder	68.82	81.23	+12.41
Kidney_L	90.97	92.87	+1.90
Kidney_R	88.60	92.72	+4.12
Liver	93.17	95.87	+2.70
Pancreas	53.24	65.78	+12.54
Spleen	90.21	93.65	+3.44
Stomach	69.13	85.50	+16.37
mDiceorgan (8 organs)	80.11	87.21	+7.10

**Table 9:** Class-wise Dice ablation results and foreground mean for eight organs on the Synapse dataset (%).

Categories	U-KAN	U-KAN-DAB	U-KAN-HMCRF	U-KAN-WPAQF	DU-KAN
Aorta	86.77	89.11	88.13	88.87	90.03
Gallbladder	68.82	63.18	70.85	71.00	81.23
Kidney_L	90.97	93.78	91.24	93.12	92.87
Kidney_R	88.60	91.00	89.18	90.90	92.72
Liver	93.17	94.49	94.73	95.01	95.87
Pancreas	53.24	56.24	59.13	58.94	65.78
Spleen	90.21	91.10	91.07	92.01	93.65
Stomach	69.13	79.68	81.38	83.16	85.50
mDice_organ_ (8 organs)	80.11	82.32	83.21	84.13	87.21

**Table 10:** Decision criteria and representative outputs for individual Oxford MEST-C indicators.

Oxford Feature	Quantitative Criteria	Representative Measurements	Representative Output
M	Mesangial hypercellularity was evaluated based on the proportion of glomeruli showing mesangial hypercellularity. Threshold: ≥50% of glomeruli with mesangial hypercellularity = M1; <50% = M0	Number of glomeruli with mesangial hypercellularity: 11/20 (55.0%)	M1
E	Endocapillary hypercellularity was classified as a binary feature. Presence of endocapillary hypercellularity = E1; absence = E0	Endocapillary hypercellularity detected: Yes (endothelial/glomerular area ratio: 1.30%)	E1
S	Segmental sclerosis was classified as a binary feature. Presence of segmental sclerosis lesions = S1; absence = S0	Segmental sclerosis detected: True	S1
T	Tubular atrophy/interstitial fibrosis was classified according to the Oxford thresholds: T0: ≤25%; T1: >25% and ≤50%; T2: >50%	Tubular atrophy/interstitial fibrosis ratio: 25.77%	T1
C	Cellular/fibrocellular crescents were classified as a binary feature. Presence of crescents = C1; absence = C0	Crescent lesions detected: True	C1

**Table 11:** Overall results of structured Oxford MEST-C determination in 40 cases.

Oxford Feature	Agreement Rate (%)	Sensitivity (%)	Specificity (%)	ROC-AUC	AP
M	85.00	62.50	100.00	0.81	0.78
E	87.50	37.50	100.00	0.69	0.50
S	87.50	76.19	100.00	0.88	0.89
T	97.50	50.00	100.00	0.75	0.53
C	85.00	70.00	90.00	0.80	0.57
MEST-C	65.00	-	-	-	-

## Data Availability

The data presented in this study are available on request from the corresponding author. The data are not publicly available due to privacy and ethical restrictions.

## Abbreviations

The following abbreviations are used in this manuscript:

IgAN	Immunoglobulin A nephropathy
PAS	Periodic acid–Schiff
MEST-C	Mesangial hypercellularity, endocapillary hypercellularity, segmental sclerosis, tubular atrophy/interstitial fibrosis, and crescents
DU-KAN	Dual-domain enhanced U-KAN
KAN	Kolmogorov–Arnold network
DAB	Dual-branch encoder
HMCRF	Heterogeneous multi-kernel coordinate recalibration fusion
WPAQF	Wavelet–prototype adaptive query fusion
WCA	Wavelet-enhanced cross-attention
PCA	Prototype-guided cross-attention
MSCM	Multi-scale channel modulation
MB-SaE	Multi-branch squeeze-and-excitation
ROI	Region of interest
GT	Ground truth
CT	Computed tomography
aAcc	Overall pixel accuracy
mIoU	Mean intersection over union
mDice	Mean Dice coefficient
ROC-AUC	Area under the receiver operating characteristic curve
AP	Average Precision
FLOPs	Floating-point operations
